# A novel STPA approach to software safety and security in autonomous maritime systems

**DOI:** 10.1016/j.heliyon.2024.e31483

**Published:** 2024-05-21

**Authors:** Alojz Gomola, Ingrid Bouwer Utne

**Affiliations:** Department of Marine Technology, NTNU, 7491 Trondheim, Norway

**Keywords:** STPA, SysML, Autonomous maritime system, Risk analysis, Software failure, Risk mitigation

## Abstract

With higher autonomy in maritime systems, tasks and responsibilities are moved from the human operator to software, increasing the complexity and the importance of safe and reliable functionality. Software failures, however, may be introduced from the early life cycle phases intentionally or unintentionally, and these must therefore be mitigated by safe and secure design approaches. A challenge is that existing methods are not particularly well-suited for analyzing software risks. Thus, the objective of this paper is to propose a systematic and efficient software failure identification approach by extending the Systems-Theoretic Process Analysis (STPA) with a software failure taxonomy and the System Modeling Language (SysML). This enables the control structure in STPA to cover both the dynamic and static aspects of the software functions. Combined with an implementation platform independent questionnaire, this gives a more systematic and guided search for potential software failures than existing approaches. To demonstrate the proposed approach, a case study on a ferry's navigation system that operates in manual control or semi-autonomous mode is performed. In the case study, the focus is on creating an avoidance map data structure, including both moving and static obstacles to be avoided by the ferry, and the subsequent process of collision risk warning calculation. Software failures are identified and evaluated in collision scenarios where the ferry operates under foggy conditions. The paper shows that the proposed systematic approach provides an improved process for identifying and analyzing critical software failures. This facilitates enhanced risk mitigation in the design and testing phases contributing to autonomous systems' safety and security.

## Acronyms

**AI**: Artificial Intelligence
**AMS**: Autonomous Maritime System
**AUV**: Autonomous Underwater Vehicle
**BPMN**: Business Process Modeling Notation
**CA**: Control Action
**CONOPS**: Concept of Operations
**CS**: Controller
**DES**: Distributed Embedded System
**DP**: Dynamic Positioning
**DPRA**: Dynamic Probabilistic Risk Assessment
**GQ**: Guiding Question
**HO**: Human Operator
**HW**: Hardware
**LoA**: Level of Autonomy
**LS**: Loss Scenario
**MASS**: Maritime Autonomous Surface Ships
**OM**: Operation mode
**RAC**: Risk Acceptance Criteria
**SF**: Software Failure
**SIS**: Software Intensive System
**SRC**: Supervisory Risk Control
**STPA**: Systems-Theoretic Process Analysis
**STPA SW SAF-SEC**: STPA Software Safety Security
**SW**: Software
**SysML**: System Modeling Language
**UCA**: Unsafe Control Action
**UML**: Unified Modeling Language
**VAR**: Variable
**VF**: Vessel Feature

## 1 Introduction

### 1.1 Background and objective

Autonomous Maritime Systems (AMS) are complex cyber - physical systems composed of hardware (HW), software (SW), and human operator (HO) parts and their interactions. The software is complementary to the HO and HW parts, and altogether they deliver the AMŚ main function in operation. A software failure may thus be a complex phenomenon that impacts the overall system's safety and security.

Evaluating hazards and potential losses related to the software functionality is challenging in current risk assessment methods due to system complexity and the constantly evolving runtime state [1], [2]. Traditional assessment tools are insufficient to ensure safe software [3], [4]. In recent years, several projects and initiatives, such as the Maritime Unmanned Navigation through Intelligence in Networks (MUNIN) [5], the Advanced Autonomous Waterborne Applications Initiative (AAWA) [6], [7], the Online Risk Management and Risk Control for Autonomous Ships (ORCAS) project [8] and IMO's Maritime Autonomous Surface Ships (MASS) [9], have shown that the software's importance and responsibilities increase with a higher level of autonomy (LoA). At the same time, the scope of the HO's tasks may decrease with an increasing LoA [10]. Software failures may also have cascading effects, meaning that they can propagate to other system parts.

Some examples of severe losses related to software hazards and failures are the recent Golden Ray ship capsizing [11] and the Boeing 737-8 MAX accidents [12], [13]. These accidents show the importance of identifying and mitigating software failures early in the design and development process to minimize the potential impact of hazards and losses in operation. Currently, however, software failures are usually found at the end of development or the beginning of deployment phases, when a significant amount of resources have been used to develop the complex system, and the cost of change is immense. This means that a hazard identification and risk mitigation approach must be feasible for systems at a high abstraction level, i.e., at an early stage in the system development process.

The objective of the present paper is to propose a novel approach to hazard analysis of software safety and the security of AMS using the failure taxonomy proposed in [14], the Systems-theoretic Process Analysis (STPA), as well as the System Modeling Language (SysML) language as a foundation. The purpose of the approach, called STPA SW-SAF-SEC, is to identify and analyze complex software failures on different system abstraction levels, complying with the following requirements:

• Model the AMS's software parts in detail, covering the dynamic software evolution in time and any changing responsibilities.
• Cover software safety and security aspects, focusing on cascading software failures.
• Evaluate the impact of software failures on the overall safety and security of the system.
• Handle risks related to software failures in AMSs by designing countermeasures.

The paper demonstrates the proposed approach in a case study focused on the navigation advisory and automation system for a semi-autonomous ferry.

The scope of the paper is to capture potential software failures occurring in an AMS that may evolve and propagate out of the softwareś system “boundary”. After reaching the software boundary, a failure may transform into a hazardous event that may lead to losses. Human errors and HW failures are not investigated in detail, as these issues are already addressed extensively in existing literature, which may be combined with the proposed approach in this paper.

### 1.2 The software functions in AMS and the development process

In software engineering, a function is a sequence of program instructions that performs a specific task, consuming the provided input and providing calculated outputs based on the inputs and the software function's internal state [15]. The software function has a public interface defining the input and output variables. This interface is used to call functions by other system parts. The invoking system parts can be the HO utilizing the user interface, hardware parts using the HW/SW interface, or other software functions.

Today, software functions may be organized in decentralized frameworks that enable asynchronous and parallel execution [16]. Software frameworks, for example, DUNE, can be deployed on swarm autonomous underwater vehicles (AUVs), as demonstrated by Pinto et al. [17]. Gezer et al. [18] developed a generic framework for AMSs based on ROS. These examples demonstrate that the abstraction level of software functions can differ from covering a specific piece of code up to complex functionality. Therefore, to ensure safety and security it is necessary to examine possible software function behaviors in the runtime environment and potential failures early, as the designated functionality is specified in the design phase.

Software functions are normally specified in the AMS's concept of operations (CONOPS), with a fundamental specification of roles and responsibilities. In the CONOPS, the software is usually modeled to a relatively high level of detail. Therefore, the software state, structure, behavior, input, and outputs should be accessible for risk assessment early in the design process. An extensive ecosystem of software modeling infrastructure supports a systematic approach to the data structures and behavioral runtime evolution in various abstraction levels. When the initial AMS is specified in the CONOPS document, the software functions may be specified in SysML. SysML (see, e.g., [19], [20], [21], [22], [23], [24]) enables definition of the software function deployment on the HW parts of the system using deployment diagrams, the software function hierarchy and relationships to other system parts through use case diagrams, the software function's dynamic runtime evolution using sequence and activity diagrams, and the software functionś discrete state evolution in state diagrams.

In the software development process of AMS, the CONOPS is transformed into a requirement specification document, which gives a greater level of detail in the form of textual functional and non-functional requirements. The requirements specification is used as one of the inputs into developing the software architecture. The software architecture separates the software functionality into modules and further into the implementation elements, utilizing Business Process Modeling Notation (BPMN), UML languages, and other software engineering approaches.

The software architecture is later transformed into the code, which ultimately is a static representation of the software function. It is important to note that software is always well documented at any abstraction level by different model views utilizing static and dynamic models covering different structural and behavioral aspects. The separation of concerns is a widely used principle in software engineering to break down and focus on specific aspects of complex systems.

For a thorough understanding of the software-related hazardous events and risks, there is a need to model the software function, including the SW-HW and SW-HO interfaces, as a white box model. Most of the software parts of the AMS can be modeled as a white box model, even though there is software related to machine learning, like neural networks, that only can be modeled as a white box up to a limited extent, which challenges the verification and validation of safety.

### 1.3 Existing risk analyses methods for software failures

Zhou et al. [25] and Johansen and Utne [26] compared different risk analysis methods for autonomous ships. Both studies concluded that STPA is the method which is able to cover the most important hazards for autonomous ships. STPA is a hazard analysis technique based on system engineering. It uses a collection of interacting “control loops” to identify hazardous events in systems, or unsafe control actions (UCA), and it may be used at any stage of the system lifecycle. STPA identifies UCAs on all abstraction levels and includes their causes in loss scenarios [4].

There are several research studies, however, combining STPA with other methods, to improve the feasibility of the analysis for the problem at hand. For example, Rokseth et al. [27] recommended using STPA and the failure mode and effects analysis (FMEA) for maritime systems. Sultana et al. [28] compared STPA and HAZOP for Liquefied Natural Gas (LNG) Ship-to-Ship (STS) transfer. Bensaci et al. [29] compared STPA with bowtie risk analysis for centralized and hierarchical architectures of mobile robots, showing that STPA is superior to bowtie risk analysis, but still recommending both methods. Sun et al. [30] applied STPA, the hazard and operability (HAZOP), FMEA, and the functional resonance analysis method (FRAM) to an automotive automatic braking systems, and showed that all methods have their advantages and disadvantages. Yang and Utne [31] analyzed an AUV operation using STPA, HAZOP and preliminary hazard analysis (PHA), and concluded that STPA provided a good basis for developing quantitative risk models, with the other methods as useful complementary tools.

STPA has been used for several unmanned and autonomous maritime vessels. Wrobel et al. [32], [33] used STPA to show the capability of STPA to identify potential failures with significant safety impacts early in the design phase [34]. The paper also concludes that there is a need to concentrate efforts on software development and validation due to uncertainties and its impact on safety performance. A challenge is to develop the control structure due to the increasing software complexity [35].

Highly connected and intelligent systems increase the system vulnerability to cyber-attacks [36]. Hence, including security-related incidents in STPA is valuable, as security is one of the main concerns in software intensive systems (SIS). Young and Leveson [37] and Friedberg et al. [38] introduced extensions of the STPA including security aspects. Zhou et al. [36] proposed an extension to STPA that synthesizes safety and security, considering different LoA for autonomous ships. Lee et al. [39] used the STPA control hierarchy for the identification of cyber security threats in automotive networks by identifying attack surfaces in systems' functions.

An STPA extension addressing multiple LoAs in autonomous underwater systems and operations was developed by Yang et al. [40]. This approach extended STPA by creating a control hierarchy for each operational mode to investigate how UCAs change with different LoA. The control hierarchies were interconnected using a state transition diagram representing the changes in LoA. The autonomous mode, however, was modeled as an “instant” transition, but for AMS operation the responsibility shift between the SW and the HO may be more gradual.

The above-mentioned approaches show that often the “classical” STPA is extended or combined with other methods to enable an improved hazard analysis of the problem at hand. A remaining challenge with the existing approaches is the limited coverage of complex software functionality. Even though, e.g., Leveson and Thomas [41] address software, there is not much guidance on how to include UCAs related to software failures. The overall STPA control hierarchy's size may, however, become intractable with the inclusion of detailed software parts into the structure, challenging the analysis of interactions and the identification of UCAs. There is also a need to improve the understanding of the software part's inner workings, which is beneficial for preventing and mitigating software failures more efficiently.

Software interacts with other parts of a system, such as HO and HW. Human errors can be identified and quantified through human reliability analysis (HRA) [42]. Thieme et al. [43] modeled the human-autonomy interaction for an AUV, focusing on HO-HW-SW at a high abstraction level. For more detailed analysis of SW-HW, for example, Diao et al. [44] present an online monitoring system that is able to detect and analyze faults. A method for analyzing HW-SW faults in the conceptual design phase is presented. Sinha et al. [45] review the literature on interaction failures of HW–SW components and conclude that existing approaches hardly consider all types of interactions. Sinha et al. [46] present a model of a hardware-software system that can be used to predict the worst case system reliability and availability in the early design phase. Such works are valuable for improving reliability of systems. A survey on software bug prioritization has been conducted by Uddin et al. [47] and their results are useful for software reliability and the late development phases. A software quality assurance method to predict software faults is presented by Moudache and Badre [48]. There is, however, a need to also focus on safety, as a reliable system is not necessarily safe [4].

A recent approach to the safe design of AMS using a concept from software engineering is the “operational envelope” [49]. This envelope may utilize the Unified Modeling Language (UML) to cover various operational scenarios and capabilities. Therefore, the proposed approach in the paper should be consistent with the concept of the operational envelope.

Krauss et al. [50] evaluated different software tools for STPA. Through the tool, SAHRA, they were able to integrate the “classical” STPA into a UML/SysML environment. The tool did not cover all software parts necessary for efficient risk assessment of complex software functions. The work shows, however, that it is possible to combine STPA with software modeling tools.

DeSouza et al. [21] combine STPA with SysML modeling activities, including simulation and formal verification. STPA is used to identify safety requirements, and SysML is then applied to structure the analysis and represent the control hierarchy. The main focus of the work, however, is on formal verification, and not on identifying software failures and potential cascading effects, related to causes and consequences. Ahlbrecht and Bertram [51] introduce model based systems engineering and present a formalization of SysML with STPA. The purpose is to allow for safety trade-offs in the early design phase. Ahlbrecht and Durak [52] expand this work and combine STPA with failure graphs to visualize the safety analysis status and coverage. These works show the benefit of combining STPA with UML/SysML approaches.

The method proposed in this paper, which we call STPA (Software) SW-SAF-SEC, therefore focuses on identifying and analyzing software failures, as this is a remaining challenge in the current works. The particular focus is on autonomous ships, and SysML is utilized to enhance the modeling of software functionality and provide a basis for the operational envelope for MASS, as suggested by Rødseth et al. [49]. A novelty is also the inclusion of a software failure taxonomy and security. The use of SysML allows for an investigation of the failures' potential cascading effects to other parts of the system. Thus, the work extends and complements existing software engineering approaches to improve the search for software failures and their effects in early-stage system design.

### 1.4 Paper organization

An overview of the STPA SW-SAF-SEC method is provided in Section 2, whereas the detailed steps are explained in Section 3. The case study showcasing the application of the STPA SW-SAF-SEC of the ferry navigation system can be found in Section 4. The results of the case study using the STPA SW-SAF-SEC approach are discussed in Section 5, followed by the conclusions in Section 6.

## 2 Overview of the STPA SW-SAF-SEC approach

**Figure 1:**
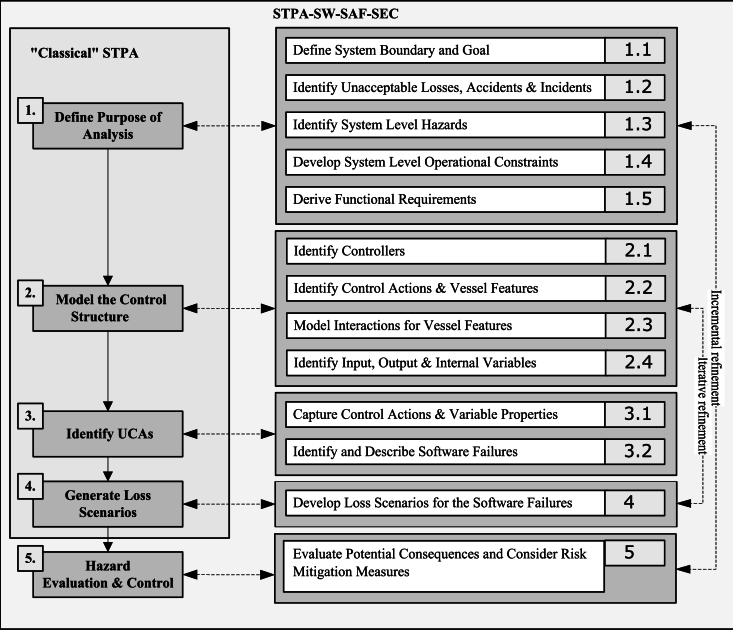
The STPA SW-SAF-SEC approach proposed in the paper, focusing on software safety and security in Autonomous Maritime Systems, adapted from Zhou et al. [36].

The first four steps of the STPA SW-SAF-SEC in Fig. 1 are structured similarly to the process of the “classical” STPA [41]. The process is inspired by Zhou et al. [36], but aims to cover software failure phenomena in particular. The purpose and scope of analysis, including the software parts, need to be specified by the user (step 1). Detailed creation of the software control hierarchy with their static and dynamic modeling (step 2) is introduced to cover the necessary level of detail for the software failure search in the static and runtime environment. To enable software reusability it is necessary to distinguish between static and dynamic modeling because the same static piece of code can be utilized in various dynamic scenarios.

In the “classical” STPA, both the dynamic and static aspects are put together in the control structure, while the internal processes of the controllers are partially hidden. For software controllers, there is a process complexity aspect that is higher than for other types of controllers (e.g., HW), which needs to be considered. The software itself is specified by static and dynamic control hierarchy models in UML and SysML notations, which gives a good foundation for inputs to dynamic analysis, such as simulation and testing.

When the control structure for the software is established, it is possible to do a guided search for initiating and cascading software failures (step 3) by applying what we call guiding questions (GQs). These questions have been developed to focus specifically on software failures more in detail than the categories of unsafe control actions (UCAs) in the “classical” STPA (too late/early/out of order, providing/not providing, stopped too soon/applied too long [41]). The software failure identification process may, however, lead to identifying UCAs in the system's operation.

Loss scenarios (step 4) are created to evaluate the global (high abstraction level) consequences of the identified potential software failures resulting in hazardous events or losses. Even though the number of potential software failures may be high, the user needs to evaluate the significance of each loss scenario's impact on the system's operation and safety. Then, elimination or mitigation countermeasures can be applied to improve safety (step 5). The effectiveness of the elimination and mitigation countermeasures can be qualitatively evaluated by utilizing the STPA control hierarchy obtained in the previous steps.

**Figure 2:**
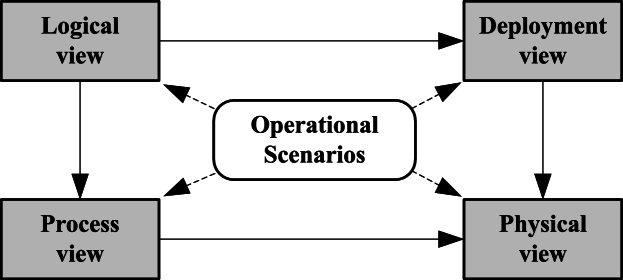
CONOPS's model views and relation to operational scenarios, based on [53].

Before starting the analysis for an AMS (“step 0”), it is necessary to have a basic overview of the CONOPS's content and logical connections. As Fig. 2 shows, the CONOPS usually contains:

1. Operational scenarios - defines expected AMS performance and operation, including both the system's operation and the environment's expected behavior; they influence the system model.
2. Logical view - defines the logical structure of system functions and capabilities, their relationships, and dependencies.
3. Process view - defines dynamic logical aspects of system functions, their time dependencies, and communication.
4. Deployment view - defines the allocation of software on physical systems, their mutual communication means, and interfaces.
5. Physical view - defines components' physical allocation and placement on AS (e.g., where we mount sensors).

Operational scenarios are reflected in the deployment view, which provides a dynamic overview of system interactions, and the logical view, which provides an overview of the static structure. The control hierarchy in the classical STPA covers both logical and process views from CONOPS.

In STPA SW-SAF-SEC, the control hierarchy from the classical STPA is extended by static (structural) and dynamic (behavioral) models taken from SysML. The reason is that software functions may be too complex to be analyzed efficiently in a single control hierarchy. Thus, in the proposed approach:

• The dynamic STPA control hierarchy refers to the dynamic SysML model extracted from operational scenarios, the process view, and the dynamic part of the deployment view.
• The static STPA control hierarchy refers to the static SysML model extracted from the deployment/physical views and the static part of the logical view.

## 3 Steps of the STPA SW-SAF-SEC approach

There are five main steps and several sub-steps in STPA SW-SAF-SEC (Fig. 1).

### 3.1 Step 1: define purpose of analysis

The first step consists of the following:

*Sub-step 1.1 - define system boundary and goal:*

The analysis starts with defining which parts of the system and its operational environment will be considered as targets for the analysis.

*Sub-step 1.2 - identify unacceptable losses, accidents and incidents:*

Then, based on the operational requirements and scope, unacceptable losses that may be caused by safety and security incidents and accidents should be identified.

*Sub-step 1.3 - identify system-level hazards:*

From the losses in the previous step, the system-level hazardous events (which are later linked to software failures and cascading effects) need to be identified.

*Sub-step 1.4 - develop system level operational constraints:*

The system's operational limits and constraints should be defined.

*Sub-step 1.5 - derive functional requirements:*

The main system functions or features, which are used as a basis for defining the vessel features (VF) (c.f. Fig. 4) later on in the analysis, should be outlined in this sub-step.

### 3.2 Step 2: model the control structure

This step covers the development of the control structure, which purpose is to model feedback and interactions to enable investigation of UCAs. It is constituted by controllers, control actions, feedback, input and outputs, and controlled processes [41] addressed in several sub-steps. In the STPA SW-SAF-SEC, the focus is on software functionality and potential interactions with HO and HW elements.

Software functionality is often implicit and encapsulated, meaning that the software functions have internal structure and interactions that the classical STPA control hierarchy does not cover. This is due to the lack of modeling tools for software in STPA, such as an internal state model, data over time evolution, and separate dynamic and data evolution mechanisms. Hence, additional details provided through static and dynamic views are added here, utilizing UML and SysML models.

UML and SysML are universal notations that can model static and dynamic properties at any system abstraction level [20]. They are widely used to model a system's functional and logical architecture. The class diagrams outline the logical partition of the software into runtime objects, whereas deployment diagrams outline the software allocation on physical systems and their communication interfaces. The deployment diagram is more suitable for a static control hierarchy as it also considers the controllers' physical allocations.

The current view of the software architecture in AMS and its relationship to HW and the HO is in line with Bucaioni and Pelliccione [54]. Regardless of implementation and deployment, the software is considered hierarchical and modular [55]. Hierarchical means a strict hierarchy of software elements with different abstraction levels. Modular means that the software is organized into enclosed units with a defined set of functions, data, and responsibilities.

*Sub-step 2.1 - identify controllers:*

The goal of this sub-step is to transform technical specifications or system knowledge into the static and dynamic views of the STPA control hierarchy that can be used for later software failure search and evaluation.

A controller is realized by a set of physical devices executing software implementation and communicating over internal and external communication lines over time. For example, the software controller for a ship is characterized by a specific set of VFs representing the software functions. The software controllers are considered a top-level static model element, encapsulating VFs and variables (VARs), and communicating with other controllers via internal or external communication networks.

The software controllers can be identified as follows:

1. Controller Identification - Identify bounded entities in deployment and class diagrams of system specifications with specific sets of functions that can be labeled with unique descriptive names.
2. Controller realization - Define how the logical controller is realized in the system. This may be based on the system's functional specification, class diagrams, deployment diagrams, etc. The controller can be characterized by the following parameters extracted from the system architecture:
  (a) Human operator - a HO realizes the controller functions.
  (b) HW - the hardware parts realize the controller functions.
  (c) Interface - the controller functions as a “bridge” from SW to HW or HO.
  (d) Software function - the controller function is realized as software implementation and can be further expanded into specific software system categories implying other properties in the analysis.
3. Type of controller - defined by the analysis boundary:
  (a) Internal controller - in the scope of the AMS design; this consists of parts under full supervision, like navigation software or off-the-shelf components with sufficient documentation.
  (b) External controller - this is partially or fully out of the scope of AMS control, like a prefabricated propulsion system or a HO with a given set of instructions following, e.g., procedures and regulations.

STPA SW-SAF-SEC focuses on software controllers of the internal type, for which a higher level of detail is available for white-box modeling. For example, the navigation software in the case study (Section 4) is regarded as a distributed embedded system (DES) and is considered an internal controller in the analysis.

The SysML deployment diagram [23] is chosen as a modeling notation for the control structure because it offers an extensive overview of system functionality. Important software interactions, often described as VF and hidden in the classical STPA, are modeled here in sequence diagrams.^1^ If necessary, the dynamic state evolution can be modeled by the employment of state transition diagrams, as has been demonstrated by Zhong et al. [56].

**Figure 3:**
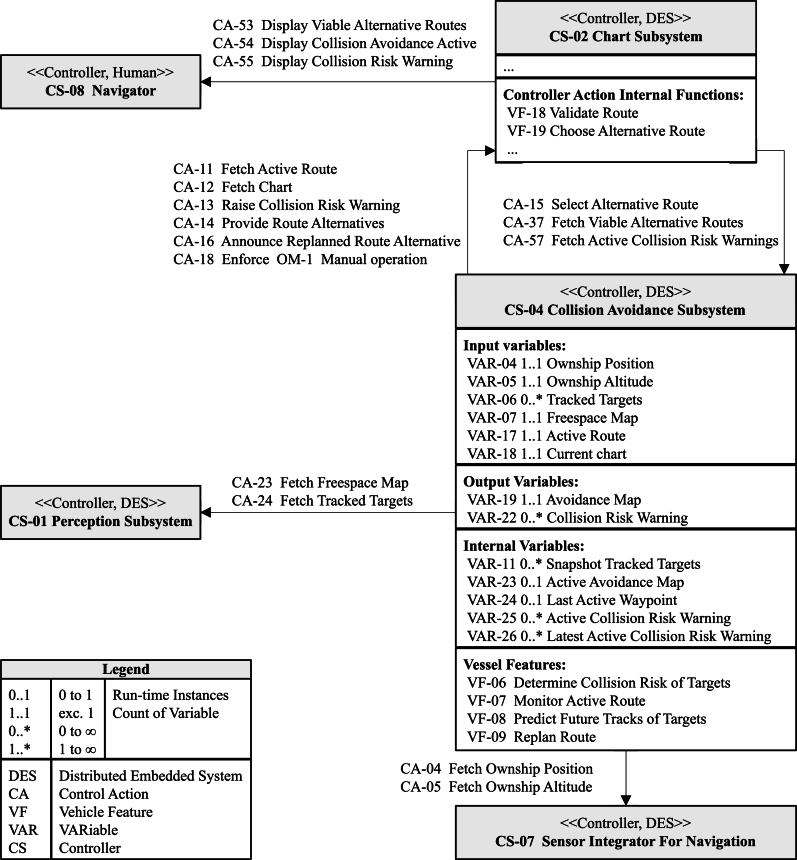
Control structure subset for the case study in the paper.

The controllers contain VARs and VFs that can realize software functions. VARs in relationship to the controller are used in the realization of VFs. The input VAR is used as input to at least one VF. The output of a VAR is used as the output of at least one VF, and the internal VAR is used in the internal processing of one VF, see Fig. 3 and, e.g., the VAR “Avoidance Map” in the case study.

Each identified controller should consist of a unique set of software functions and VARs. If the system design is truly modular, this may be considered as an implicit property. If the system design is not truly modular, it is up to the user to define controllers and create a mapping table of their relationships.

*Sub-step 2.2 - identify control actions & vessel features:*

This sub-step aims to identify control actions (CAs) and VFs and assess each responsible controller identified in the previous sub-step. Controllers, their VARs, and CAs have a static model that defines their allocation which resembles the traditional control hierarchy in the classical STPA. The software function is strongly context- and execution-dependent and modeled as an extension in the STPA SW-SAF-SEC approach to include the dynamic aspects. This extension is novel for the proposed approach and important for software safety and security.

The VF is a bounded software function that can be described via UML and SysML activity diagrams, sequence diagrams, or textual functional specifications. The VF, by definition, is a dynamic interaction activity resulting in output VARs or CAs. The VF is invoked by, contains, and may result in CAs. A CA is a time-bounded action targeting a specific controller with its data payload, expecting execution of an action or a reply data payload from the controller. A CA can be mapped to an activity or sequence in the SysML model or represent multiple activities bounded by time and data. Identifying CAs and VFs is an iterative process.

The VFs and CAs can be extracted as follows:

1. Determine a VF that belongs exclusively to the controller and has a sufficient description of internal processing in the system documentation.
2. Determine the VF's properties, if possible:
  (a) Level of control, i.e., automatic, conditional, manual, etc. This describes by whose authority the VF is executed.
  (b) Runtime execution, i.e., periodical or on demand. This determines when the VF is executed.
3. Examine the VF's description and identify CAs in function calls or out-of-system boundary communication.

VFs are important for the identification of software failures and recovery, level of control, and their runtime execution defines periodicity and execution conditions. There is the possibility of identifying duplicate CAs for different VFs. Then additional functional analysis and the creation of a general listing are recommended means to prevent this.

CAs are used in the dynamic part of the STPA control hierarchy to show communication with other controllers in the SysML sequence diagrams. The result of this sub-step is the preliminary listing and allocation of VFs and CAs to the controllers.

*Sub-step 2.3 - model interactions for vessel features:*

This sub-step aims to model the dynamic aspect of the software controller, which is one of the novelties of the approach of this paper. In this step, it is necessary to capture the time and data flow in the dynamic model of the controller's VF using the SysML sequence diagrams. For practical purposes, SysML sequence diagrams capture all necessary information in one model, as they are easy to read and understand for outside field experts. The sequence diagram consists of lifelines representing “system parts,” which potentially can be HO, HW, or SW controllers. It is possible to model internal parts of software components, like persistent and volatile memory, where VARs are stored.

The time-sensitive dynamics of the model is captured by the messages sent between the various system parts in sequence or parallel. The sequence diagram also captures the conditionality of each VF's execution, enabling the modeling of alternative flows. The VF's dynamic model uses a static part of the STPA control hierarchy as a basis for the relevant system parts, and is created in multiple iterations. At the first iteration, when the VFs and CAs for all controllers are identified, the following steps should be followed to create the sequence diagrams:

1. Identify the system's parts in the interactions. This can be extracted from the VF's functional description, depending on the abstraction level.
2. Annotate the system's parts according to their properties. The necessary level of detail is annotated on the system parts, physical location, type, and parameters. Annotations are derived from the CONOPS and the results of step 1.
3. Create an initializing event, which is the first message from the initializing system parts that activates the sequence of messages, e.g., for periodical VFs, a scheduled function run.
4. Model the main flow of actions and events from the functional description, i.e., model data exchange, modification, internal function, and the CAś calls.
5. Map and unify the identified CAs. This means that the CAs identified in sub-step 2.2, which calls controllers outside the VFś scope should be consistently formulated.
6. Model alternative flows of actions and events, i.e., modeling conditional parts of the activity sequence with condition specifications.

In the later iterations, when the controller VARs are consolidated (sub-step 2.4), it is necessary to map each of the VAR's dynamic flow into existing sequences. It is further recommended to map and refine each CA's interaction. The result of this sub-step is dynamic models for each VF in the form of SysML sequence diagrams.

*Sub-step 2.4 - identify input, output & internal variables:*

This sub-step aims to consolidate the VARs over the static (deployment diagram) and dynamic (sequence diagrams) control hierarchy created in steps 2.1-2.3. The VARs for the VFs are obtained through the functional decomposition of use cases describing their functionality. The output is a consolidated VAR table. The VARs represent data used to support the controller's intended function, and they have dynamic properties of the time validity, data consistency, and correctness.

The VAR's time validity means that the VAR is valid for a specific timestamp or time period, e.g., an AIS message is valid for a few minutes from receiving it. A VAR's consistency means a quality guarantee over a defined time period or that a snapshot evolution between the same VARs shall have a similar level of data quality, e.g., the deviation of the static obstacle positions shall not vary much.

A VAR's correctness describes the structure data correctness, or how well the data represent the state of the natural world. The data structure is by default valid or has minor acceptable deviations, e.g., the tracked vessel's position does not deviate too much. Moreover, VARs have static implementation properties which can be used in the software failure identification:

1. Data Type - the data type is delivered from the functional specification, influenced by technical terms or platforms, on the system's abstraction level. Two significant types are defined:
  (a) Data stream - a stream of data that changes over time, e.g., camera or radar readings.
  (b) Structure - a system-defined structure with discrete creation time and meaning of every field in the structure. This can be further specified as the abstraction level decreases, e.g., AIS messages and, collision avoidance warnings.
2. Origin - defines the controller reference, where data originates, are created or are changed/entered for the first time into the system. This can be an external or specific controller. VARs are reused in multiple controllers, and therefore the controller that changes or creates variables impacts the evolution of software failures.
3. Time Sensitivity - defines if the VAR is time sensitive. If the VAR changes over time, it can cause synchronization or behavioral software failures.
4. Description - description of the VAR's content or behavior, extracted from the functional specification/use case.

It is also important to distinguish between a VAR's definition and instance during the analysis. A controller's ownership, time sensitivity, and VFs characterize a VAR's instance, e.g., the radar reading in the perception controller is different from the VAR's instance radar reading in the display subsystem.

CAs may be similar to the VARs in their dynamic behavior, having a definition consisting of an action, a target controller, and execution consisting of the context, VARs, and VF context. As CAs represent function calls and communication between software systems, the execution context evolves with time, e.g., reading radar data returns different VARs if it is called from different controllers at different times. The result of this step is a list of VARs with a description of their content and system allocation, see Table 4.

### 3.3 Step 3: identify unsafe control actions

This step covers the identification of potential software failures, their causes, consequences, how to trace them, and their recoverability. A software failure leads to an alteration of software function runtime execution that can cause additional cascading software failures, ultimately resulting in propagation out of the SW part of the AMS and the software failure's manifestation in the form of UCAs, hazardous events, and losses. The outcome of this step is a list of software failures that may cascade into potential losses.

*Sub-step 3.1 - capture control actions & variables' properties:*

This sub-step addresses the context for the identification of potential software failures, focuses on selecting critical and dependable elements of the STPA control hierarchy, and reducing the scope of the identification of software failure. Not all software functions may necessarily contribute to software safety amd security, and software functions are usually separated into safety critical and non-safety critical parts. Hence, the user may want to reduce the scope of the analysis, which could be done by focusing on the following:

1. Identify the critical safety and security control flows - in the software part of the system, there are observable functions, e.g., displaying a collision risk warning, that contribute to the overall system safety and security, which should be identified. These observable software function is realized as a runtime execution of VFs consisting of VARs and CAs.
2. Identify contributing CAs - in the critical control flow, there are CAs that have a different level of influence on the software's safe and secure execution. For example, proper delivery of collision risk warnings is critical for safe obstacle avoidance; therefore, it is necessary to identify related CAs.
3. Identify dependable variables - in the critical control flow, VARs are processed inside dynamic VF models. The VARs that are related to runtime evolution, data quality, or sequence that can impact the safety and security of the process are dependable VARs.

*Sub-step 3.2 - identify and describe software failures:*

This sub-step aims to find possible software failures, here focusing on the identified safety and security critical control flow. It is recommended to start with VFs that are at the beginning of the critical control flow and continue until the top controller is reached. The process is guided by an iterative, incremental search for software failures, in which the STPA control hierarchy developed in step 2 can be used as a basis. To ensure completeness, the identification of the software failures is structured by using guiding questions (GQs) on the identified controllers, VFs, and CAs from the previous steps. The GQs are applied one by one to a selected element of the STPA control hierarchy. The GQs are shown in Table 1. These have been developed based on experience with software for distributed embedded systems (DES). The questions cover the software failure categories, as follows:

1. Resource exhaustion (REX) - this takes into account the software resource structure in computation, memory, and storage. The software function has evolving demands during its execution in the runtime environment, which is dependent on situation. Therefore, the resource requirements should be scaled within the available physical resources.
2. Behavioral (BEH) - the software function's behavior can be different from design expectations even when data are delivered on time and in sufficient quality. The intended behavior is crucial for safe software execution and result delivery.
3. Synchronization (SYN) - the software's function is executed in a timely and sequential manner, i.e., it can be on-demand or periodical. Uf there are time-sensitive VARs, the possible effect of their time validity needs to be examined.
4. Data Degradation (DDE) - VARs are obtained via various means such as communication exchange, loading from persistent storage, or retrieval from volatile memory. The qualitative properties of VARs may fluctuate. It is necessary to examine if the possible change of quality in VARs can impact the runtime execution of software functions.
5. Configuration (CON) - the system configuration changes and evolves over deployment time due to such factors as the replacement of HW components, the update of software components, or a change in HO procedures. External systems and their operation can also change over time. It is necessary to identify sensitive configuration aspects that can negatively impact software function.
6. Security Related (SEC) - the system can be exploited by an insider/outsider actor who wants to gain unlawful access or cause destruction to the system or software function. It is necessary to identify possible security vulnerabilities and attack surfaces that the attacker can exploit.
7. Cascade (CAS) - software failure is not an isolated event. Software failures can be triggered via a cause-consequences relationship. Therefore, it is necessary to examine if a software failure can cause undesired changes in other controllers and VFs, ultimately leading to UCAs, hazardous events, and losses.

**Table 1:** Guiding Questions (GQ) for the software failure search in a System Controller Model.

Cat.	ID	Applicable to:			Question	Failure/Effect
		[CA]	[VF]	[CS]		
REX	GQ-01	Yes	Yes	Yes	Is there a variable which needs to be scaled?	Volatile memory exhaustion
	GQ-02	Yes	Yes	Yes	Is there heavy mathematical calculation processing?	Computation power exhaustion
	GQ-03	Yes	Yes	No	Is there persistent data modification?	Persistent storage exhaustion
	GQ-04	Yes	Yes	No	Is there a run-time variable that is changed often?	Memory fragmentation
	GQ-05	Yes	Yes	Yes	Is there usage of HWI or HMI with high throughput demand?	HWI/HMI overrun
	GQ-06	No	Yes	Yes	Is there a greedy element that shares parallel resources?	Concurrent resource exhaustion

BEH	GQ-07	No	Yes	Yes	Are processed variables mutually exclusive?	Multiple sources of the truth
	GQ-08	Yes	Yes	No	Are variable constraints well defined?	Uncovered variable combinations
	GQ-09	No	Yes	Yes	Are conditional execution of features mutually exclusive?	Overlapping constraints
	GQ-10	Yes	Yes	Yes	Is there a situation that can lead to a false positive action execution?	False positive behavior
	GQ-11	Yes	Yes	Yes	Is there a situation that can lead to a false negative action execution?	False negative behavior
	GQ-12	No	Yes	No	Is there unwanted behavior for any combination of variable ranges?	Undefined state space behavior
	GQ-13	Yes	Yes	Yes	Can something influence intended behavior?	Function-specific behavior failure

SYN	GQ-14	Yes	Yes	No	Is there a variable or action accessed (near) real-time?	Lags, timeout, interrupt, retry failures
	GQ-15	Yes	Yes	No	Is there a variable where time validity impacts function execution?	Lags, timeout, retry failures
	GQ-16	Yes	Yes	No	Is there usage of snapshots action execution?	Invalid and outdated variable snapshot
	GQ-17	Yes	Yes	No	Is there an interrupt causing an atomicity violation?	Atomicity violation
	GQ-18	No	Yes	Yes	Is it possible to execute an action too soon, too late, or not in the right order?	Atomicity violation, order violation
	GQ-19	No	No	Yes	Is there synchronization of emerging functionality?	Multiple contributors synchronization

DDE	GQ-20	Yes	Yes	No	Is there a variable or action accessed online?	Network error and aging failures
	GQ-21	Yes	Yes	No	Is there a variable serving as a counter, unique identifier, or iterator?	Value overflow failure
	GQ-22	Yes	Yes	No	Is there a variable stored in persistent storage and loaded into memory?	R/W operations from persistent storage
	GQ-23	Yes	Yes	Yes	Are there multiple instances of a variable in volatile/persistent memory?	Value corruption and validity failures
	GQ-24	Yes	Yes	No	Is an action maintaining multiple mirrors of same variable?	Multiple sources of truth failure
	GQ-25	Yes	Yes	Yes	Are missing or malformed static variables checked over time?	Data degradation of static variables

CON	GQ-26	Yes	Yes	No	Is there a variable sourced from an external system which is out of direct system control?	Integrated variable failures
	GQ-27	Yes	Yes	No	Is there part of an action execution depending on an external system?	Integrated functionality failures
	GQ-28	Yes	Yes	No	Is an internal system part necessary for changeable action execution?	Software update-related failures
	GQ-29	Yes	Yes	Yes	Is there a configuration change over lifetime influencing action execution?	Configuration change failures

SEC	GQ-30	Yes	Yes	Yes	Is there a possibility of action exploitation to gain any advantage?	Exploitable data and features
	GQ-31	Yes	Yes	Yes	Is there any residual attack surface the attacker can exploit?	Asset vulnerability
	GQ-32	Yes	Yes	Yes	Is any variable transferred over an exposed communication channel?	Open or unsecured communication
	GQ-33	Yes	Yes	Yes	Is there a variable that can be exploited via manipulation to change an action execution?	Data and flow manipulation

CAS	GQ-34	Yes	Yes	Yes	Is there an existing software failure which can be propagated out?	Producing cascading failures
	GQ-35	Yes	Yes	Yes	Is there an existing underlying software failure which can pass through unhandled?	Propagating cascading failures
	GQ-36	Yes	Yes	Yes	Is there an existing underlying software failure that can be transformed into hazard/loss?	Transforming cascade failures into hazard/loss
	GQ-37	Yes	Yes	Yes	Is a system or environment-specific issue causing cascade failure?	Domain-specific cascade failures

Further description about the categories and taxonomy can be found in Gomola et al. [14]. The GQs are formulated to give a binary answer. Then, a more detailed analysis is necessary, with respect to their cause, trace, consequences, and recoverability:

**Cause:** When a positive answer to a GQ is triggered, there is a possibility of software failure. The STPA control hierarchy can be used to determine the cause, which is dependent on the software failure type. For initiating software failures (GQ-01 to GQ-33), the cause is a random or intended change of the examined element. Initiating software failures may lead to one or more cascading software failures. For cascading software failures (GQ-34 to GQ-37), the cause can be the previous initiating or or other cascading software failures in the event chain. A cascading software failure may be caused by multiple underlying software failures in different scenarios.

**Trace:** A software failure impacts the runtime behavior of the software function modeled in the static and dynamic parts of the STPA control hierarchy. The trace examines which CAs and VARs in underlying VFs or controllers that are impacted by the failure event. The trace is created by following the dynamic evolution of changed elements over the functional scope. If the trace is inside a sequence diagram, it follows the control flow until the end of the VF is reached. If the software failure is inside a controller, it follows the control flow until the boundary of the controller is reached. If the software architecture defines interface boundaries, the trace will follow the dynamic evolution until the defined boundary. During the trace evaluation, UCAs and hazardous events may be triggered, which may become consequences of a software failure.

**Consequences:** The consequences of software failures are collected as a list of deviations from a run without failure. In the case of cascading software failures, the list also includes the consequences of all preceding software failures in the event chain. The consequences listed by the trace also include changed or malformed VARs that are considered output or internal state VARs of the examined STPA control element. Formulations of UCAs may be user-defined, depending on the implementation platform and abstraction level. “Degradation” of VAR's is software failure dependent, and the terminology of synchronization, data degradation, and integration of software failures' malformities can be used. The formulation of consequences should be related to the system-level hazards identified in step 1. Whereas the losses may affect the controller functionality at a high abstraction level, the consequences are more specific to the software failure.

**Recoverability:** This is dependent on the cause, trace and consequence of the software failure. The general rule of thumb is that an automatically and periodically executed VF has a higher likelihood of recovering if further propagation of the software failure is mitigated. For example, if a wrong sensor reading is causing data degradation, the software failure can be recovered on the next sensor reading if the data are not malformed.

STPA-SW-SAF-SEC can be combined with “classical” STPA. This means that the GQs should be used for identifying software failures, and then the “standard” STPA categories for UCAs [41] could be applied to the control hierarchy for identifying UCAs related to human performance, HW, and interactions.

### 3.4 Step 4: generate loss scenarios

In this step the software failures from step 3 that may evolve into system losses or hazards in the system operation, should be organized into loss scenarios. The loss scenarios are created in a similar manner as in the classical STPA, but a difference is having the detailed event description of potential software failures from step 3; with causes, consequences, trace, and recoverability. This additional information may contribute to finding interconnected software failures via cascading effects with cause-consequence relations. The operational conditions and system state influences a software failure's event initialization, cascading effect, and propagation over system parts.

The loss scenario creation process uses the STPA control hierarchy from step 2 and the identified potential software failures from step 3. As the software failure may propagate to the HW-SW or SW-HO interface, mapping software controllers to their deployment locations may be advantageous. The following tasks suffice as a guideline when develop the loss scenarios:

1. Determine loss scenario preconditions, including the internal VAR's state of controllers prior to the software failure's event introduction.
2. Select an initiating software failure and analyze the impact of the software failure's event by using the STPA control hierarchy. This may rely on the information already gathered on cause, trace, consequence, and recoverability.
3. Compare causes/consequences of the identified potential software failures in an iterative process. If there is an existing cascading software failure, link it; if there is a new cascading software failure, create a new one according to sub-step 3.2.
4. Continue repeating task 3, until the software boundary is reached or until software failure is diminished (e.g., the system recovers from the software failure, but the software failure does not propagate over the system boundary).
5. When the SW-HW or SW-HO interface is crossed, continue with “standard” loss scenario generation in STPA.

Simulation can be a good tool for investigating how and where software failures may manifest itself. This can be done as suggested, e.g., in Sinha et al. [46], or for generating test cases for verification of safety critical and software-intensive systems, such as [57], [58], [59].

When all loss scenarios are created, the user should select the software failures for which mitigation measures need to be considered in step 5.

### 3.5 Step 5: hazard evaluation & control

This step aims to reduce the risk of the identified potential software failures by their elimination and mitigation. The ultimate goal is to improve the safety and security of software parts by cost efficient improvements in the system's early design. Since STPA is a qualitative analysis, the potential consequences, and losses are decisive for determining the need for risk reduction measures. Safety goals or qualitative risk acceptance criteria (RAC) may be relevant to consider to be able to evaluate if the countermeasures are sufficient. Safety goals or RAC are determined by the stakeholders involved, and related to their corresponding goals, as well as relevant regulations.

Briefly, the relevant software failure elimination and mitigation techniques can be categorized as follow:

1. Preemptive countermeasures that aim to prevent software failures by design, which is a traditional approach focusing on software reliability.
2. Permissive countermeasures that aim to eliminate the consequences of software failure with extensive testing.
3. Fallback procedures that aim to define software function behavior in case of software failure, usually in the form of a backup algorithm, that guarantees system operation under the effects of software failure. An example is a deterministic calculation of an obstacle avoidance trajectory that guarantees a suboptimal but safe trajectory, or a sensor fusion method that is capable of calculating values when a single sensor fails.
4. Operational constraints aim to define performance limitations to prevent losses from hazardous events that may result from software failures, e.g., preventing the system from operating in autonomous mode in an environment with low visibility.

## 4 Case study - analysis and results

The case study in this paper focuses on a real roll-on/roll-off ferry with a capacity of 600 passengers and 200 cars operating in Norway between two terminals as a pendulum ferry. The purpose of the case study is to demonstrate the approach presented in Section 3. The information in the case study has been anonymized in agreement with industry partners.

The ferry crossing takes approximately 25 minutes at an average speed of 12 knots. The operation consists of pre-departure procedures like loading fuel, passengers, cars, and unmooring. When the crew loads and checks the ferry, the departure phase from port starts. During the departure phase, the ferry is steered manually by the navigator under the master's supervision. After the ferry leaves the harbor area, it follows one of the preprogrammed paths to the target harbor. This is called the transit phase, which is executed or supported by the “MarCrew” system.

The ferry can operate in manual or semi-autonomous (LoA2) transit modes. In the manual mode, traffic warnings are provided and alternative routes are suggested to the navigator on the bridge. In the autonomous transit mode, the ferry navigates a selected route and requires the navigator's approval for a route change whenever needed. When the ferry approaches the harbor, it enters the arrival phase. It is then docked automatically or manually by the navigator. The HO in the case study is the crew onboard the ship.

The case study focuses on the transit phase, where the MarCrew system plays a critical role in ensuring operational safety and security. The MarCrew system provides two critical operational capabilities:

• “Maintain lookout,” which provides an enhanced overview of the ferry's surroundings and operational area to the navigator by the utilization of onboard sensor systems; and
• “Navigate route,” which provides the crew with navigational advisories or autonomous navigation functions depending on the ferry's operational mode.

The case study is based on the ferry's operational conditions and the system design outlined in the concept of operations (CONOPS).

### 4.1 Step 1: define purpose of analysis

*Sub-step 1.1: define system boundary and goal*

The case study focuses on the collision avoidance functionality as a subset of the MarCrew system. The goal is to identify potential software failures leading to increased operational hazards or unacceptable losses. The MarCrew system software outlines the boundary on the input side by a sensor array consisting of radar and camera systems providing data to the electronic chart subsystem utilizing HO/SW interactions on the output side. The sensor system and navigator are considered external actors, and only internal MarCrew functions are considered here, i.e., operational capabilities for lookout, navigation support with collision avoidance, and automation. In normal weather conditions, the transit has good visibility and constant crew supervision. The ferry's technical state, crew condition, and other outside factors are considered acceptable by existing ferry operation standards.

*Sub-step 1.2: identify unacceptable losses, accidents & incidents*

The MarCrew provides navigational advisories or automation under the assumption that only accidents and incidents related to inland waterway transits, where commercial traffic passes, as well as leisure vessels are considered. The traffic implies risk of collision. There is a shallow shore nearby one of the terminals, which also creates a risk of the ferry grounding. Hence, the potential loss of life and injuries to the crew and passengers, as well as third-party stakeholders are relevant. In addition, property loss and damage may be expected due to the terrain, ferry speed, and the traffic in the operational area.

*Sub-step 1.3: identify system-level hazards*

Hazards leading to losses can be caused by intentional third-party actions (security) or unintentional random events (safety). Both types of hazards are considered in the case study. The list of safety and security hazards, modified from [60], [61], leading to the unacceptable loss of life or property is as follows:

1. Collision - the ferry is on an unavoidable collision track with another ship, or moving object.
2. Allision - the ferry is on an unavoidable collision track with stationary floating or submerged objects, including static obstacles (bridges) in the waterway.
3. Grounding - the ferry is on an unavoidable grounding track to a shore/cliff.
4. Misconduct - the ferry is transmitting erroneous information to other ships, endangering the safety of navigation/lookout.
5. False positive/negative MarCrew decision - the surrounding situation is not correctly determined, for example, objects are not detected and recognized correctly (small objects, navigation marks, ships, ship lights, floating objects, depth, recreational craft, people) or the weather is not correctly perceived (wrong/no output).
6. Data theft - third parties steal and misuse data from the ferry sensor system.
7. Control overtake - the ferry is navigating to another destination port other than the desired one, due to piracy, hijacking, or stealing.

*Sub-step 1.4: develop system-level operational constraints*

The CONOPS defines system-level constraints for the intended MarCrew usage. For example, the MarCrew shall be used only during ferry transit in the manual or semi-autonomous mode, depending on the navigator's decision. If MarCrew is in the autonomous transit mode and an “unsolvable” situation occurs, or the navigator's response is significantly delayed, the system shall switch to the manual mode and forward responsibility to the HO (navigator). The lookout functions to display ship surroundings and enhanced sensor data are used exclusively by the navigator as a supporting process. The environmental constraints for the MarCrew system are a stable sea state (wave height <1.5 m, wind speed <20 knots) and good visibility >0.5 nm.

*Sub-step 1.5: derive functional requirements*

The functional requirements related to collision avoidance, which are the basis for the VFs, are the MarCrew's capability to provide the following functions (complying with the ferry's operational constraints):

1. Detection, classification, and target tracking for moving and static obstacles.
2. Prediction of the future track of targets.
3. Determination of collision risk with moving and static obstacles.
4. Route monitoring and replanning in case of increased collision risk from tracked targets.

### 4.2 Step 2: model control structure

The control structure is derived from MarCrew's CONOPS. The critical focus is white box modeling of the designed software functions on the system's abstraction level using an iterative identification of each controller's properties. The MarCrew model, for all operational capabilities, consists of 9 controllers representing software intensive systems (SISs), sensors, and HOs. In total, 22 interactions were modeled representing VFs. 34 VARs were captured covering instances of internal, input, and output data structures, and 57 CAs were identified representing external interactions between the controllers. Only a subset of functionality for collision avoidance and navigation is showcased here in the case study (Fig. 3).

*Sub-step 2.1: identify controllers*

The controllers have been identified and mapped according to the CONOPS deployment SysML diagram, and their properties are summarized in Table 2. The identification was a deductive analysis of the VF realizations (shown in the sequence diagrams in Figure 5, Figure 6). As a result, the identified controllers are as follows:

• Perception Subsystem - this is an internal controller realized as a DES, processing sensors' readings into identified tracked targets which are processed later by the collision avoidance system.
• Chart Subsystem - this is an internal controller realized as a DES with an HO-SW interface, processing collision risk warnings from the collision avoidance subsystem and communicating with the navigator.
• Collision Avoidance Subsystem - this is an internal controller realized as a DES, processing the received situational assessment into collision risk warnings and constantly evaluating “collision risk” along the journey.
• Sensor Integrator For Navigation - this is an external controller realized as a DES, serving as input to the navigation capability and providing data for the perception processing function.
• Navigator - this is an external controller representing a HO interacting with MarCrew during the transit phase to receive traffic advisories or monitor semi-autonomous execution.

**Table 2:** Subset of the Identified Controllers.

ID	Name	Realization	Type
CS-01	Perception Subsystem	DES	Internal
CS-02	Chart Subsystem	DES	Internal
CS-04	Collision Avoidance Subsystem	DES	Internal
CS-07	Sensor Integrator For Navigation (SINT) Subsystem	DES	External
CS-08	Navigator	HO	External

Each controller is deployed on a separate embedded computer with or without a display unit exchanging data over a common asynchronous bus. The HO realizes the navigator's role, and therefore, no further mapping is done here.

The controllers' placement in the control hierarchy is shown in Fig. 3 (logical view) and reflects their authority and hierarchy. The first level consists of the navigator and Chart Subsystem controllers, responsible for decision-making during the transit phase. The second level consists of the Perception and Collision Subsystem controllers responsible for decision support by providing a situation overview and collision risk warnings. The CAs, VARs, and VFs are only shown here for the Collision Avoidance Subsystem controller. In the case study, the full control hierarchy was used for the identification of potential software failures, but for illustration, only the important controller related VFs, CAs, and VARs are shown here.

*Sub-step 2.2: identify control actions & vessel features*

**Figure 4:**
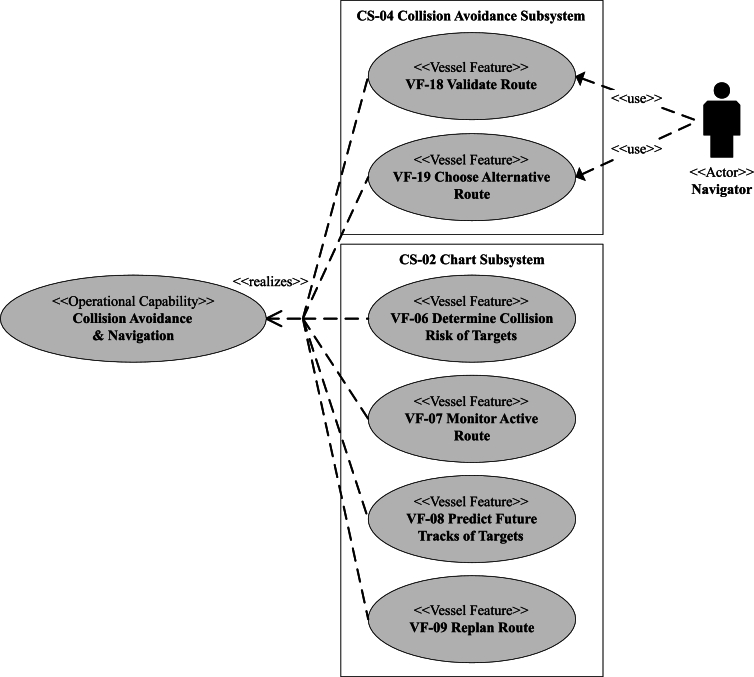
The functional decomposition of navigation and collision avoidance operational capabilities for the case study in the paper, based on the CONOPS.

The MarCrew's navigational capability is decomposed into VFs in CONOPS. Each VF contains a functional description in the form of a use case, covering the ideal main and major alternative scenarios. Navigational capability is realized by VFs that are deployed on a different system, for example, by the VFs shown in Fig. 4. In addition, one VF can realize multiple operational capabilities and some VFs include HO (navigator) interaction over the user interface.

**Figure 5:**
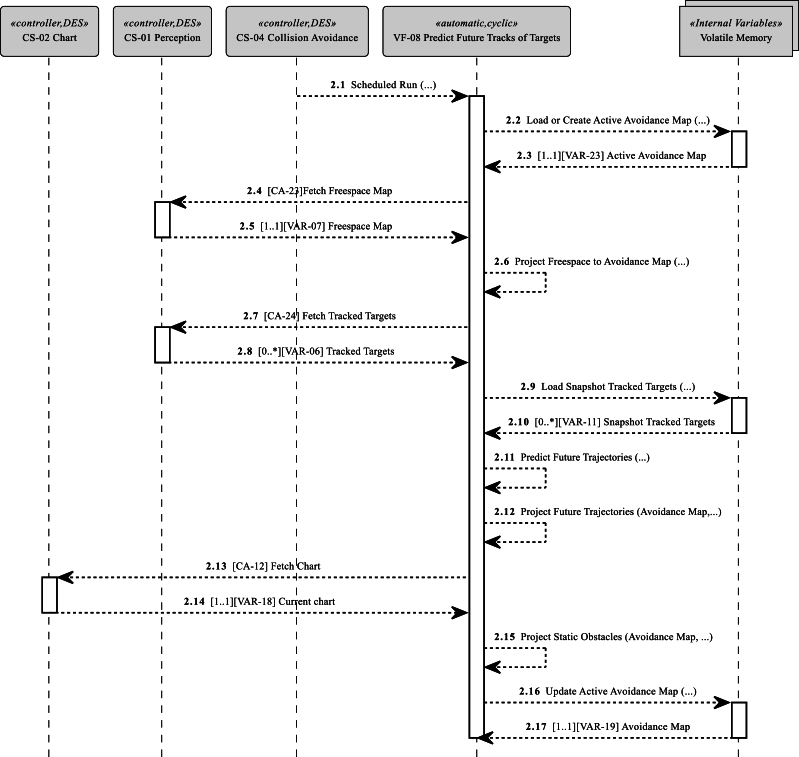
Vessel Feature [VF-08] Predict Future Tracks of Targets sequence in case study.

**Figure 6:**
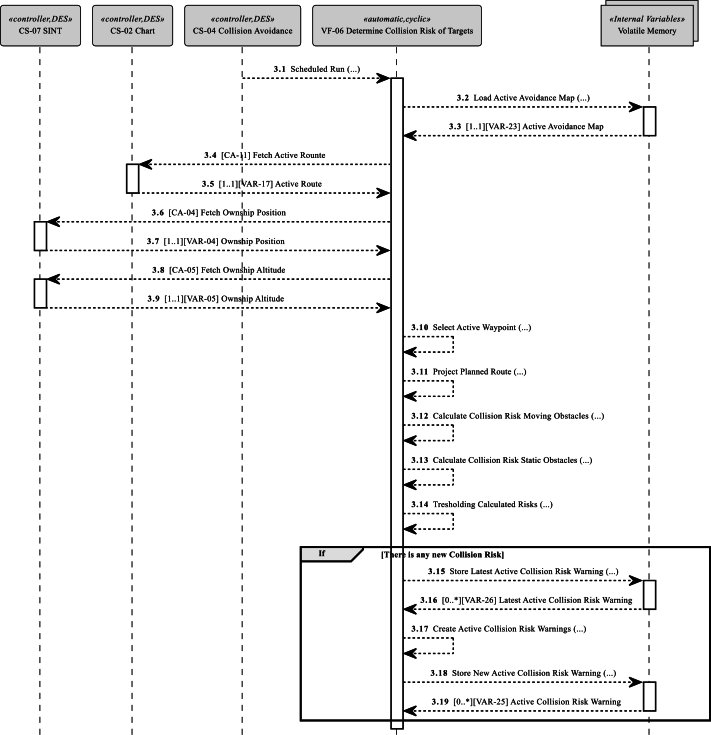
Vessel Feature [VF-06] Determine Collision Risk of Targets sequence in case study.

Two VFs are the subject of this case study: Firstly, Predict Future Tracks of Targets (VF-08 in Fig. 5), which predicts future movements of moving obstacles and results in the creation of a VAR-19 avoidance map (Table 4). An avoidance map represents the current collision risk situation in the operational ferry area and is a critical component for navigational decision-making. Secondly, Determine the Collision risk of Targets (VF-06 in Fig. 6), which processes an avoidance situation and creates a list of VAR-22 Collision Risk Warnings to be further processed by the navigator.

CAs are identified as actions in the functional description of VFs that target other controllers or their own controller's internal structure with an action verb. In the first iteration, the basic CAs are extracted. Software design promotes reusability, and therefore, there is a high likelihood of finding duplicate CAs with similar functionality targeting the same controllers. In consecutive iterations, the identified CAs have been unified into the final list of CAs in Table 3. The identified CAs represent HO/SW interaction, pull/push data requests, and active enforcement on other controllers. Each CA has its target controller, and different controllers can invoke it.

**Table 3:** Subset of the Identified Control Actions.

ID	Contol Action	Target	Functional description
CA-04	Fetch Ownship Position	[CS-07]	Retrieves the latest [VAR-04] Own ship position from [CS-07]
CA-05	Fetch Ownship Altitude	[CS-07]	Retrieves the latest [VAR-05] Own ship altitude from [CS-07]
CA-11	Fetch Active Route	[CS-02]	Retrieves actual [VAR-17] Active and [VAR-21] Active Waypoint from [CS-02]
CA-12	Fetch Chart	[CS-02]	Retrieves actual [VAR-18] Current chart from [CS-02]
CA-13	Raise Collision Risk Warning	[CS-02]	Raise collision risk warning to [CS-02]
CA-14	Provide Route Alternatives	[CS-02]	Provides [0..*] [VAR-20] Alternative Route to [CS-02]
CA-15	Select Alternative Route	[CS-04]	Replies with [0..1][VAR-20] Alternative Route or Timeout to [CS-04]
CA-16	Announce Replaned Route Alternative	[CS-02]	Announces new [1..1][VAR-20] Alternative Route to [CS-02]
CA-18	Enforce [OM-1] Manual operation	[CS-02]	When the selection of [VAR-20] Alternative Route fails during raised Collision Risk, [OM-1] Manual Operation is enforced on [CS-02]
CA-23	Fetch Freespace Map	[CS-01]	Retrieves actual [VAR-07] Freespace Map from [CS-02]
CA-24	Fetch Tracked Targets	[CS-01]	Retrieves actual [VAR-06] Tracked Targets from [CS-02]
CA-37	Fetch Viable Alternative Routes	[CS-04]	Requests viable alternative routes according to current obstacle avoidance restrictions
CA-53	Display Viable Alternative Routes	[CS-08]	Alternative routes during collision risk warning view are displayed to the navigator
CA-54	Display Collision Avoidance Active	[CS-08]	A collision Avoidance warning is displayed to the navigator
CA-55	Display Collision Risk Warning	[CS-08]	A collision Risk warning is displayed to the navigator
CA-57	Fetch Active Collision Risk Warnings	[CS-04]	Fetch Active Collision Risk Warning from [CS-04]

*Sub-step 2.3: model interactions for vessel features*

In the case study, two VFs for safety-critical collision avoidance functionality are modeled as sequence diagrams based on MarCrew CONOPS functional description and the previously identified CAs. The control hierarchy's SysML diagrams (Figure 3, Figure 5, Figure 6) are created on the system's abstraction level and focus on external controller communication and data flow. The sequence diagrams show a time-lapse of CA calls, internal processing, and projections into the avoidance map. The controller's scheduler periodically invokes VF. The ownership of the controllers and VF are given by the control structure (Fig. 3), where VFs belonging to specific controllers are listed, and the controller ownership and role can be assigned.

VF-08 Predict Future Tracks of Targets (Fig. 5) obtains actual obstacle avoidance situations from the electronic chart and perception subsystems and processes them into a prediction of the future tracks of adversaries and positions of static obstacles.

VF-06 Determine Collision Risk of Targets (Fig. 6) is periodically invoked by the controller's scheduler. Its main function is to evaluate the ferry's safety on the active route and generate VAR-25 Active Collision Risk Warnings (Table 4). The VF is critical in decision-making because it triggers automatic obstacle avoidance behavior in the semi-autonomous transit mode or warns the navigator about possible collisions in manual mode. Any failure of this or preceding functions may lead to collisions.

**Table 4:** Subset of the Identified Variables.

ID	Variable Name	Origin	Time Sensitive	Variable Description
VAR-04	Own ship Position	[CS-07]	Yes	Position of the ship in the local and global coordinate frame, valid for a specific time
VAR-05	Own ship Altitude	[CS-07]	Yes	Roll/pitch/yaw in the local and global coordinate frame, valid for a specific time
VAR-06	Tracked Targets	[CS-01]	Yes	Data of tracked targets, ID, position, heading, and velocity, gathered over the time period
VAR-07	Freespace Map	[CS-01]	Yes	Representation of operational space valid for a specific time and own ship's position
VAR-11	Snapshot Tracked Targets	[CS-01]	Yes	Historical tracked targets with timestamp
VAR-17	Active Route	[CS-02]	Yes	Active navigational path monitored over [CS-02] executed by the ferry, a set of waypoints used during automatic transition
VAR-18	Current chart	[CS-02]	Yes	Current digital chart of the operational area
VAR-19	Avoidance Map	[CS-04]	Yes	Combination of Freespace Map, Predicted Routes, Map Obstacles, and Sensed Obstacles for determination of the safe operational area
VAR-20	Alternative Route	[CS-04]	No	Set of waypoints in the operational area usable during the transit operation phase
VAR-21	Active Waypoint	[CS-02]	Yes	The current navigational goal during the transit operation phase belonging to [VAR-17] Active Route
VAR-22	Collision Risk Warning	[CS-04]	Yes	Indication of Collision Risk Warning, including tracked target or map obstacles and collision probability
VAR-23	Active Avoidance Map	[CS-04]	Yes	Active working avoidance map
VAR-24	Last Active Waypoint	[CS-04]	Yes	Latest Active Waypoint
VAR-25	Active Collision Risk Warning	[CS-04]	Yes	Active Collision Risk Warnings, containing collision objects, risk of collision, and time to collision
VAR-26	Latest Active Collision Risk Warning	[CS-04]	Yes	Previous Collision Risk Warnings used as a reference for collision risk calculations

Note: All showcased variables have Structure Data Type				

VAR-23 Active Avoidance Map representing the current situation in the ferry's operational area, is used as the basis for collision risk evaluation. The collision probability for each tracked target, map obstacle, and detected static obstacle along the active route is calculated. The calculations for all possible collision targets are compared with the limit values. If the calculated collision risk exceeds the limit value, the Chart Subsystem and navigator later process the active VAR-22 Collision Risk Warnings (Table 4) in decision-making.

*Sub-step 2.4: identify input, output & internal variables*

The VF's functional decomposition results in the control and data flow being captured in sequence diagrams. The sequence diagrams identify major data structures or streams processed in the VFś realization. On the system's abstraction level, they are given symbolic names representing the content of the data structure. The identified VARs are summarized in Table 4.

The ownership of a VAR is defined in Table 4 as the origin property. The owning control structure creates and maintains the actual mirror of the variable, while the other control structures may maintain the VAR as an input/output/internal VAR (see Fig. 3). The ownership of CAs is defined in Table 3 as target property. The control structure “implements” a CA and “provides” a public interface, and the call of the CA is shown as an oriented arrow in Figure 3, Figure 5, Figure 6.

As an example, VAR-06 Tracked Targets (Table 4) is a VAR representing the set of the detected moving obstacles, originated in the Perception Subsystem, where it is created and retrieved via CA-24 Fetch Tracked Targets (Table 3) in VF-08 Predict Future Tracks of Target (Fig. 5). There are two different instances of the tracked target VAR (Fig. 5): First is a real-time snapshot which serves as input into the Collision Avoidance Subsystem. Second is Snapshot Tracked Targets, the internal VAR of the previously retrieved Tracked Targets with different time validity also used in VF Predict Future Tracks of the Targets. The emphasis on the time aspect is strong, as almost all VARs are time sensitive and change their content during system operation. The only exception is the VAR-20 Alternative Routes' (Table 4) set of preprogrammed routes that are set before ferry operation begins.

### 4.3 Step 3: identify unsafe control actions

The critical software functions and failures related to creating collision risk warnings are the further focus here.

*Sub-step 3.1: capture control actions & variables properties*

The MarCrew's STPA control hierarchy (Figure 3, Figure 5, Figure 6) need to be scrutinized to identify the critical CA and VAR properties, which again provide a basis for identifying software failures and UCAs in the next step (and later loss scenarios). Here, the identification is made as a top-down analysis of the control flow from the navigator controller as the starting point and up to the Perception Subsystem as the endpoint, given by the system boundary (defined in step 1).

The identification of the safety and security critical control flow starts at the STPA control hierarchy (Fig. 3), where the navigator interacts with the Chart Subsystem to process an alternative route selection in case of a Collision Risk Warning. The Chart Subsystem is the monitoring system where two critical VFs contribute to navigational capability: VF-18 Validate Route is executed periodically and implemented as an internal procedure. This VF constantly checks for sailed route safety and relies on the provided VAR-22 Collision Risk Warnings. If VF-18 Validate Route detects a new collision risk that is unavoidable in the future, it on demand invokes VF-19 Choose Alternative Route. VF-19 Choose Alternative Route utilizes HO-SW interaction with the navigator, and its decision is forwarded to the Collision Avoidance Subsystem. VF-19 over CA-15 Select Alternative route invokes on-demand VF-09 Replan Route from the Collision Avoidance Subsystem.

The Collision Avoidance Subsystem periodically runs the following VFs in sequence: VF-08 Predict Future Tracks of Targets, VF-06 Determine the Collision Risk of Targets, and VF-07 Monitor Active Route. VF-07 Monitor Route checks new VAR-22 Collision Risk Warnings if there is an impact on the safety of VAR-17 Active Route. If the conditions are met, the CA-13 Raise Collision Risk warning is invoked on the Chart Subsystem. VF-06 Determine Collision Risk of Targets is responsible for calculating a set of the VAR-22 Collision Risk Warnings based on the operational situation and VAR-19 Avoidance Map. VAR 18 Avoidance Map is created during the periodical run of VF-08 Predict Future Tracks of Targets, where input VARs from other systems are processed in collision risk calculations for static and moving obstacles.

The further analysis focuses on VF-06 Determine Collision Risk of Targets, which is the entry point in the critical control flow related to collision avoidance.

The identification of contributing CAs and depending VARs starts at the STPA control hierarchy's sequence models for VF-08 Predict Future Tracks of Targets (see Fig. 5). The sequence of CAs was followed for the CONOPS's main scenario and included an evaluation of the CAs' impact on safety and security:

• VAR-23 Active Avoidance Map is loaded from the Collision Avoidance Subsystem's volatile memory at the beginning, continuously manipulated during VF's runtime, and stored as VAR-19 Avoidance Map at the end.
• CA-23 Fetch Freespace Map retrieves VAR-07 Freespace Map from the Perception Subsystem.
• VAR-07 Freespace Map contains information on actual detected static obstacles, limitations, and boundaries; it is projected onto VAR-23 Active Avoidance Map.
• CA-24 Fetch Tracked Targets retrieves VAR-06 Tracked Targets from the Perception Subsystem.
• VAR-06 Tracked Targets contains information on actual moving obstacles and parameters necessary for their future path predictions.
• VAR-11 Snapshot Tracked Targets, containing historical snapshots of VAR-06, is loaded from the volatile memory of the Collision Avoidance Subsystem.
• Both actual (VAR-06) and snapshots (VAR-11) impact the precision of the calculation of restricted areas in the VAR-23 Active Avoidance Map.
• CA-12 Fetch Chart retrieves VAR-18 Current Chart from the Chart Subsystem.
• VAR-18 Current Chart contains static obstacles charted in the operational area.

Static map obstacles and static detected obstacles are later projected onto VAR-23 Active Avoidance Map. Each mentioned CA retrieves necessary data for the final VAR-19 Avoidance Map calculation, identified as a core VAR in the critical control flow. Each of the above-mentioned VAR influences precision, time, and space validity in the VAR-19 Avoidance Map and can influence the runtime execution of subsequent VFs in the critical control flow.

*Sub-step 3.2: identify and describe potential software failures*

The identification of potential software failures in the case study follows the whole safety and security critical control flow defined in sub-step 3.1. The GQs proposed in Table 1 were applied iteratively to the VFs in the Collision Avoidance Subsystem (Figure 5, Figure 6).

As a result, 19 initiating software failures, SF-01 to SF-19, were identified for VF-08 Predict Future Tracks of Targets (Fig. 5). Five cascading software failures, SF-20 to SF-24, caused by initiating software failures were identified for VF-06 Determine Collision Risk of Targets (Fig. 6) in the context of the control structure (Fig. 3).

Positive answers to GQs give a “causal outline” that has been further analyzed into fully described software failure events, including UCAs, that are presented in Table A.9 in the Appendix. A summary of the software failures (SF) is found below:

• Resource Exhaustion (REX). SF-01 to SF-04 are caused by bad VAR scaling or inefficient underlying trajectories and obstacle projection algorithms, with consequences of imprecise, omitted, or delayed results delivery.
• Behavioral (BEH). SF-05 to SF-08 are caused by malformed data structures for detected static, map, and moving obstacles coming from other controllers or the volatile memory of the Collision Avoidance Subsystem, with the consequence of false positive or false negative detection of moving or static obstacles.
• Synchronization (SYN). SF-09, and SF-10 are caused by wrong time consistency or time invalidity of stored VARs used in the trajectory prediction off moving obstacles, with consequence of inconsistent future tracks of target prediction.
• Data Degradation (DDE). SF-11 to SF-14 are caused by data degradation of input VARs retrieved via CAs from other subsystems, namely VAR-07 Freespace Map, VAR-06 Tracked Targets, and VAR-18 Current Chart, with the consequence of an invalid VAR-19 Avoidance Map incorporating incorrect risk predictions for the ferry's operational area.
• Configuration (CON). SF-15 to SF-17 are caused by HW and SW changes in controllers providing VARs over CAs for VAR-19 Avoidance map calculations, with the consequence of permanent function loss or significant degradation of quality of VAR-19 Avoidance Map.
• Security Related (SEC). SF-18, and SF-19 are caused by maliciously input VARs with the intention of disabling the MarCrew system or gaining an advantage, with the consequences of a valid, correct, but inconsistent VAR-19 Avoidance Map.
• Cascade (CAS). SF-20 to SF-24 are caused by initiating SF-01 to SF-19, which changes the state of the internal VAR-19 Avoidance Map. VF-06 Determine Collision Risk of Targets processes the VAR-19 Avoidance Map to create VAR-25 Active Collision Risk Warnings, representing possible collision threats. First, SF-20 Unavailable Avoidance Map leads to ultimate navigational capability loss if it does not recover. Second, SF-21 and SF-22, where a nonexistent obstacle is considered a threat, may lead to the hazardous events of collision, allision, and grounding. Third, SF-23 and SF-24, where an existing obstacle is omitted and not treated as a possible collision threat, may lead to hazardous events of collision, allision, and grounding.

**Recoverability:** In the case study, both the examined VFs are automatic and have scheduled runs planned by the Collision Avoidance Subsystem Controller. Hence, it may be assumed that they are running with sufficient frequency and that they are designed in a way to automatically recover in the next run (see Table A.9 “Recoverability”). Recoverability for almost all initiating software failures is possible if the initial cause is removed in the next scheduled periodical run of the VF. Configuration (CON) and Security Related (SEC) software failures are not recoverable due to the permanent presence of underlying causes. Recoverability of Cascading (CAS) software failures is possible if the initial initiating software failure recovers in the next scheduled run of the VF.

### 4.4 Step 4: generate loss scenarios

The initiating software failures SF-01 to SF-19 from step 3 (Table A.9) can be used as initializing events in the loss scenarios to examine if they lead to hazards or losses. In addition, MarCrew's operational constraints have been used as a guideline. One resulting loss scenario is shown in Table 5 for SF-07 Omitted Moving Obstacle Trajectory prediction, including relevant information from Table A.9.

**Table 5:** Collision Loss Scenario overview in the case study.

Loss Scenario: Moving Obstacle Collision due to Unmitigated Software Failure	
**Preconditions:**	
1. Ferry is on a collision course with a moving obstacle.	
2. Navigator is unable to maintain a lookout function and is completely dependent on the system due to thick autumn/spring fog in the operational area.	
3. MarCrew is unable to enforce the manual transit rule despite an operation constraint breach related to visibility (<0.5 nm)	
4. MarCrew is in automatic transit mode (semi-autonomous LoA-2)	
5. Unmitigated SF-07 [BEH] Omitted Moving Obstacle trajectory projection in VF-08	

**LS-01.** [SF-07][VF-08][S.2.12][BEH] Omitted Moving Obstacle trajectory projection	

**LS-02**. [SF-23][VF-06][S.3.2][CAS] Omitted moving obstacle in [VF-06]	

**LS-03.** [VF-07][S.6.2][CAS] Valid, Incorrect Collision Risk Warnings processed	
**Cause:**	[LS-02] Valid, Incorrect [VAR-25] Active Collision Risk Warning
**Trace:**	1. [VF-07][S.7.2] Check Triggers (...) is evaluated as negative for moving obstacle collision
	2. [VF-07][S.8.1] Invoke VF-09 Replan Route (...) is not executed due to a false negative trigger from [VF-07][S.7.2]
**Consequences:**	1. [UCA][CA-20] Monitor Active Route failed - failed detection of moving obstacles
	2. [UCA][CA-22]Replan Route is not invoked due to false negative triggers
**Recoverability:**	Yes - If [VF-06] recovers

**LS-04**. [VF-09][S.0.1][CAS] Not invoked by [VF-07]	
**Cause:**	[LS-03]
**Trace:**	1. [VF-09][S.4.3][CA-13] Raise Collision Risk Warning - not invoked
	2. [VF-09][S.9.1][CA-18] Enforce [OM-1] Manual operation - not invoked
**Consequences:**	1. [UCA][CA-13] Raise Collision Risk Warning - not invoked
	2. [UCA][CA-18] Enforce Manual operation - failsafe procedure failed
**Recoverability:**	Yes - If [VF-07] recovers

**LS-05.** [VF-18][S.2.1][CAS] No Collision Risk Warnings for Moving Obstacles fetched from memory	
**Cause:**	[LS-04]
**Trace:**	[VF-18][S.4.2] Invoke [VF-19] Choose Alternative Route not invoked
**Consequence:**	[UCA][CA-50] Choose Alternative Route - not invoked due to false negative trigger
**Recoverability:**	Yes - If [VF-07] recovers

**LS-06.** [VF-19][CAS] Not invoked by [VF-18]	
**Cause:**	[LS-05]
**Trace:**	1. [CA-53] Display Viable Alternative Routes - not invoked
	2. [CA-54] Display Collision Avoidance Active - not invoked
	3. [CA-55] Display Collision Risk Warning - not invoked
**Consequences**:	[LS-07],[LS-09]
**Recoverability:**	Yes - if [VF-07] recovers before [LS-10] (point of no return) is reached

**LS-07**. [HAZARD][SYSTEM] No Moving Obstacle Warning displayed	
**Cause:**	[LS-06]
**Trace:**	Navigator is not warned about the possible collision risk
**Consequence:**	The ferry is on a collision path
**Recoverability:**	Yes - if [VF-07] recovers before [LS-10] (point of no return) is reached

**LS-08.** [HAZARD][NAVIGATOR] No Moving Obstacle Detected at lookout	
**Cause:**	Navigator is unable to pay sufficient attention
**Trace:**	Possible collision is not detected by the navigator's perception
**Consequence:**	There will be no action from the navigator
**Recoverability:**	No - the navigator's detection ability is impaired by thick fog

**LS-09.** [HAZARD][SYSTEM] Ferry operates in [OM-2] auto routing [OP-3]	
**Cause:**	[LS-06]
**Trace:**	Ferry follows Active Waypoint leading to the collision
**Consequence:**	Ferry stays on a collision path
**Recoverability:**	Yes - if [VF-07] recovers before [LS-10] (point of no return) is reached

**LS-10.** [EVENT] Moving Obstacle Unavoidable (point of no return)	
**Causes:**	1. [LS-07],[LS-08],[LS-09]
	2. Moving obstacle too close
	3. Ferry does not have any remaining maneuvering capability
**Consequences:**	1. Ferry no longer has the maneuvering capability to avoid moving obstacle
	2. Moving obstacles cannot avoid ferry
	3. System does not recover from failure

**LS-11.** [LOSS] Collision with Moving Obstacle	
**Cause:**	[LS-10]
**Consequences:**	1. Ferry collided with Moving Obstacle
	2. System is in the failure state

The navigation capability of MarCrew in the semi-autonomous transit mode is examined under heavy operational conditions. A fundamental precondition here is that the ferry is on a collision course with a moving obstacle, e.g., a cargo ship or leisure watercraft. The navigator cannot maintain manual lookout capability and fully relies on the MarCrew system, which means that the operational visibility constraints are violated for the system.

Under these preconditions, SF-07 (Table A.9) occurs, and it does not recover because the recoverability conditions cannot be met (LS-01). The runtime state of the STPA control hierarchy is changed from the regular operation into a failure state, which results in SF-23 Omitted Moving Obstacle in VF-06 (Table A.9.) and LS-02. For example, S.2.12 refers to the corresponding step in Fig. 5 and BEH (behavioral) to the software failure category. The software failure continues cascading over controllers in the loss scenario steps from LS-03 to LS-06, with corresponding causes, traces, CAs and UCAs, until the software boundary controller reaches the navigator's HO-SW interface.

The direct consequence is that the navigator is not warned about a possible collision (LS-07), which is considered a hazard under the loss scenario preconditions. Two other hazardous events may also occur simultaneously: First, the lookout (HO) may not detect the first moving obstacle in question (LS-08). Second, the ferry may not switch into manual operation mode (LS-09) as it should normally. The ferry may continue navigating to the next waypoint in an unnoticed failure state until the point of no return on a collision course is reached (LS-10). The consequence is then a collision with a moving obstacle in due time (LS-11) which is considered an unacceptable loss according to the loss analysis from step 1. In this context, any type of collision is considered unacceptable; hence, a moving obstacle can be any type of ship (cargo ship, tanker, leisure craft etc.).

For loss scenarios exceeding the boundary of the control structure and propagating to other systems used in the example (LS-07 to LS-11), a refinement of the causes and consequences should be performed to reveal more details. The SF-07 and SF-23 are considered critical software failures because they may lead to collision, and they need to be handled in step 5.

In general, all the identified potential software failures (Table A.9) may be critical as they may cascade into possible system losses. The majority of the software failures are recoverable, which indicates a robust design of the MarCrew software. The causes are related to data time integrity, precision, and quality. The scalability of trajectory projection functionality and subsequent collision warning calculation possess a minor issue, as both VFs are designed for up to 100 moving obstacles (tracked targets). If the operational environment of the ferry is changed to a more cluttered one, the significance of resource exhaustion may increase.

### 4.5 Step 5: hazard evaluation & control

The basic idea is to prepare qualitative countermeasures like a testing strategy or design changes that minimize the impact of potential software failures, mitigate the occurrence of a software failure, or define under which circumstances a software failure is acceptable. The results presented in this section compare the traditional and progressive approaches to software failure handling. MarCrew is non-deterministic, distributed, and often utilizes heuristic searches to achieve operational navigation capability.

The list of proposed hazard mitigation measures are included in Table 6, related to the different software failures and loss scenarios developed in the case study (Table A.9, Table 5). The software failures have been evaluated in the context of the MarCrew's operation, based on their consequences, causes, traces, recoverability, and software failure type. Then potential countermeasures have been developed based on the following principles:

1. Preemptive and permissive countermeasures were mapped based on trace and software failure type; examples are provided for initiating (VF-08) and cascading (VF-06) software failures.
2. Fallback procedures were developed based on the fault tolerance approach from EN 50128 [62] and MarCrew's CONOPS.
3. Operational constraints were developed based on MarCrew's CONOPS and system engineering methods.

**Table 6:** Hazard mitigation of the identified software failures.

Software failure	Leading to hazard/losses	Preemptive countermeasure(s)	Permissive countermeasures	Fallback procedure	Operational constraint(s)
[SF-01] [SF-02] [SF-03]	[VF-08] Function degradation, [CS-02] function loss, [FERRY] collision	Priority queuing	Stress testing, resource usage testing	Deterministic fixed memory and capped complexity implementation of [VF-08]	Define limits for [VAR-06] Tracked Targets changes and [VF-08] calculation complexity

[SF-04]	[CS-02] Function degradation, [FERRY] allision, [FERRY] collision	Analysis of common limit conditions	Scenario-based testing, Monte-Carlo testing	Merge [VAR-24] Tracked Targets with similar properties to satisfy [CS-02] limits	Display heavy traffic warnings, decrease cruising speed, and increase safety margins

[SF-05] [SF-06]	[VF-08] False positive result, [CS-02] misconduct, [FERRY] grounding	Analysis of sources of dependent failures, analysis of common limit conditions	Monte-Carlo testing, fault injection testing, scenario-based testing	Using multiple [VAR-18] Current Chart and [VAR-06] Tracked Targets snapshots to determine the validity and consistency of current data.	Decrease cruising speed if detection of obstacles is unreliable

[SF-07] [SF-08]	[VF-08] False negative result, [CS-02] misconduct, [FERRY] collision	Analysis of sources of dependent failures, error guessing	Requirement-based testing, fault injection testing, scenario-based testing	Using multiple [VAR-18] Current Chart and [VAR-06] Tracked Targets snapshots to determine the validity and consistency of current data.	Increase safety margins based on current cruising speed to enable [VF-08] recovery after failure

[SF-09] [SF-10]	[VF-08] Function degradation, [CS-02] function degradation, [FERRY] collision, [FERRY] grounding	Time-invariant design, asynchronous modular arbiter	Synchronization monitoring	Enforce OM-1 manual mode if obstacle avoidance capability is lost	Increased frequency of requesting, [VAR-07] Freespace Map, [VAR-06] Tracked Targets, [VAR-18] Current chart

[SF-11] [SF-12] [SF-13] [SF-14]	[VF-08] Function loss, [CS-02] function loss, [FERRY] collision, [FERRY] grounding	Robust design, value guarding	Communication testing, Communication monitoring	Enforce OM-1 manual mode if obstacle avoidance capability is lost	Increased frequency of requesting and consistency checking of [VAR-07] Freespace Map, [VAR-06] Tracked Targets, [VAR-18] Current chart

[SF-15] [SF-16] [SF-17]	[VF-08] Function loss, [CS-02] function loss	Equivalence of classes for HW/SW integration, analysis of external and internal interfaces	Back-to-Back testing, internal and external interface testing, Interface consistency check	Enforce component compatibility check-up procedure on each system cold start to verify minimal operational requirements	Define compatible software update policies, HW upgrade, and maintenance policies, and define version compatibility pools

[SF-18] [SF-19]	[VF-08] Misconduct, [CS-02] misconduct, [FERRY] control overtake, [FERRY] collision	Application of ISO/IEC TS 19249:2017, encoded & encrypted communication design	Interface resistance testing, penetration testing, ethical (white hat) hacking	2oo2 implementation (function doubling), the voting algorithm for safety-critical decisions (obstacle avoidance capability)	Enable operation of the system only when no security breaches are detected by surrounding and own software

[SF-20]	[VF-06] Function loss, [CS-02] function loss, [FERRY] collision, [FERRY] grounding	Analysis of functional dependencies, HIL/SIL simulations	Functional doubling	Enforce OM-1 manual mode if obstacle avoidance capability is lost	Display obstacle avoidance failure warning

[SF-21] [SF-22]	[VF-06] False positive result, [CS-02] misconduct, [FERRY] grounding	Analysis of functional dependencies, defensive programming	Real-life condition testing, long-term testing	Consistency check between own [VF-06] snapshots of [VAR-24] Tracked Targets with retrieved [VAR-19] Avoidance Map	Increase safety margins based on current cruising speed to enable [VF-06] recovery after failure

[SF-23] [SF-24]	[VF-06] False negative result, [CS-02] misconduct, [FERRY] collision	Analysis of functional dependencies, defensive programming	Real-life condition testing, long-term testing	Consistency check between own [VF-06] snapshots of [VAR-24] Tracked Targets with retrieved [VAR-19] Avoidance Map	Decrease cruising speed if detection of obstacles is unreliable

Software failures related to computational power and memory exhaustion (SF-01 to SF-03) may be grouped as they have the same consequences leading to VF-08 degradation, CS-02 function loss, and may lead to a possible ferry collision with a static obstacle. Moreover, they all start at the same entry point and use similar resources. Their causes are different, but it is more cost-efficient to handle software failures at their bottlenecks.

The preemptive countermeasure is to use priority queuing, which is a design change in resource allocation and control that gives priority to input processing based on significance. Permissive countermeasures focus on testing both computational limits and memory limits on various input data sets. This guarantees that the VF is able to handle resource demands under normal operational circumstances and defines the VF's limits after development.

The fallback procedure is realized as the backup implementation with memory and computational power caps, giving safe results if the main algorithm fails and a software failure is detected.

Operational constraints are designed to limit the input size of VAR and the VF's computation complexity in MarCrew's functional requirements. In general, resource exhaustion can be handled by preemptive and permissive countermeasures, but improved safety may be achieved by also implementing a fallback procedure.

Data degradation software failures on input VARs to VF-08 (SF-11 to SF-14, see Table A.9) lead to VF-08 function loss and CS-02 function loss, resulting in possible ferry grounding or collision in the MarCrew's automatic transit mode. Moreover, all data originates in different controllers and is transmitted over a shared communication bus.

The preemptive countermeasure is to introduce robust data design with value guarding that will ensure that the input data are valid, correct, and consistent. A permissive countermeasure is to enforce communication testing after development on top of the communication monitoring when MarCrew is in operation. The fallback procedure is based on the assumption that if the MarCrew is unable to provide operational navigation capability and the ferry operates in the automatic transit mode, then it is the navigator's responsibility to take over and provide the navigation operational capability. This is assured by invoking manual mode on autopilot, which also handles communication with the navigator.

If the input VARs in question are unreliable regarding data quality, it is necessary to increase their request frequency from other systems. Preemptive and permissive countermeasures cannot fully eliminate data degradation software failures. Operational constraints may ensure data quality and mitigate the likelihood of potential software failure to a reasonably acceptable level, as it can also be a part of the general design policy and be supported directly by the communication protocol. The fallback procedure eliminates the data degradation software failures, but at the cost of shortening the operational time of MarCrew's automatic transit mode, as most fallback procedures force automatic transit mode into the manual mode.

## 5 Discussion

This section is structured into five topics:

1. Implications of the STPA SW-SAF-SEC approach
2. Comparing the case study results with other STPA analyses for validation
3. Compatibility of STPA SW-SAF-SEC with other STPA approaches
4. Applying the results of the STPA SW-SAF-SEC approach to quantitative risk modeling
5. Limitations and further work

### 5.1 Implications of the STPA SW-SAF-SEC approach

The maritime industry is driven by rapid technological transition. Remote control, automation, and internet of things may lead to increased efficiency of ships and ports. Still human supervision will be critical, supported by AI to perform tasks in the future [63]. Such technologies are software-intensive, which underscores the need for risk analysis methods enabling efficient identification and prevention of software failures. According to [64], the complexity of software systems, and uncertainty related to machine learning and AI-based systems may lead to severe consequences, such as loss of property and fatalities. Cyber-safety and cyber-security are emphasized as the main sources to new risks. The novel approach in this paper, focusing on both software safety and security may therefore help mitigating such risks.

The approach is expected to be useful for different types of stakeholders, such as risk analysts working with software intensive systems, industry developing software-intensive and safety critical systems, researchers working with AI and ML, as well as policy makers focusing on rules and regulations of such technologies. The level of detail in the analysis may be determined by the user, depending on the purpose and goal. The results of the proposed method may contribute to the whole team's better understanding of the software's inner workings because it provides the whole picture of software cooperation and collaboration with other parts of the system development process.

In the case study in this paper, 24 software failures were identified. These were identified using GQs to an extended control hierarchy, and analyzed with respect to causes, consequences, trace, and recoverability. The resulting information was included in loss scenarios. The loss scenarios consist of event sequences with the identified software failures leading to hazardous events, further resulting in losses. Since the focus in STPA SW-SAF-SEC is on software failures, no UCA directly related to the HO or HW was identified. The GQs presented in this paper are viable as standalone artifacts for good safety and security coding practices and can be used on any system level of abstraction or software development tool.

Design decisions impact the economic costs increasingly during the system development life cycle. Hence, it is beneficial to consider efficient risk reduction and countermeasures already in the early design phase. The classical STPA enables hazard analysis in the early life cycle, which is thus also provided by STPA SW-SAF-SEC in this paper. The proposed approach has been tested in a case study, which shows that the STPA SW-SAF-SEC can be applied to incomplete software specifications (CONOPS), even on the system and operational abstraction levels.

When it comes to hazard mitigation in terms of implementing fallback procedures these are usually designed to handle single software failures and retain normal nominal software functionality. A relevant fallback procedure in the case study may be to force a switch from automatic to manual transit mode, i.e., switching control from the autonomous navigation system to the human operator. A solution like this poses higher demand on the navigator's capability and skills to handle hazardous situations since monitoring may lead to boredom and the time to gain sufficient situational awareness and react properly may be limited. A risk-based decision-making support model for offshore dynamic positioning (DP) operations was examined by Hogenboom et al. [65], and studies show that human operators of DP systems may have significant difficulties when a sudden switch from automatic to manual mode occurs. Such solutions or fallback procedures must therefore be carefully considered and designed to ensure sufficient safety.

It is normally more (cost-) efficient to implement risk reduction measures early in the chain of events. An example related to the case study is that it may be better to fix faulty radar readings by the implementation of an alternative filtering method, then fix the tracked targets projection, rather than calculating an alternative avoidance map VAR with higher computation cost. Enforcing operational constraints is an alternative in late system development stages, where significant failures can be prevented by constraining the system's operation according to findings from the loss scenarios. For example, the loss scenario (Table 5) points out that the ferry cannot be operated if visibility is insufficient. Therefore, software failures may be prevented by ensuring that system operation occurs in good visibility conditions.

Simulation (e.g., Hardware-in-the-loop (HIL) and Software-in-the-loop (SIL)) is often used in testing, validation, and verification. The resulting list of identified software failures when using the proposed approach may also be utilized as input while creating test cases or validation checklists (see, e.g., [57]).

### 5.2 Comparing the case study results with other STPA analyses for validation

A key differentiator of STPA SW-SAF-SEC is an improved capability to model software parts using advanced modeling techniques from UML and SysML. Compared to the classical STPA approach, where only controllers and CAs are considered without VF interactions and VARs, the STPA-SW-SAF-SEC increases the fidelity necessary for enabling more efficient identification of potential software failures. Since the runtime evolution of the inner software function workings and data structures is explicitly defined, it is possible to use the GQs for an exhaustive analysis of potential software failures. This provides much more details related to software failures, than by applying the conventional UCA categories in the “classical” STPA (too late/early/out of order, providing/not providing, stopped too soon/applied too long). The GQs, however, is not meant to entirely replace the UCA categories; rather, they provide a supplement.

Comparing with results from a classical STPA focused on an autonomous ship [66], including the navigation system, and with similar losses, i.e., collision (allision) and grounding accidents, Table 7 shows some typical results for comparison, focusing on collision avoidance. Furthermore, Table 8 presents relevant results from [67] focusing on autonomous navigation. Whereas the former is focused on the technical systems onboard, the latter STPA is focused on human-autonomy collaboration in shore control centers.

**Table 7:** Some results from a “classical” STPA addressing collision (allision) and grounding accidents for autonomous navigation, from [66].

ID	Controller	CA	UCA description
UCA1	Model predictive control (MPC)	Calculate trajectory on horizon	The calculated risk levels along the predicted trajectory is unacceptable.
UCA2	Model predictive control (MPC)	Calculate trajectory on horizon	The predicted trajectory returned by the MPC directly causes an obstacle allision or grounding.
UCA7	Nonlinear programming (NLP)	Compute obstacle distances	The returned distances to obstacles are incorrect producing an inaccurate/unsafe basis for the trajectory calculations, leading to UCA1/UCA2.
UCA8	Non linear programming (NLP)	Compute obstacle distances	The geometric operations or distance calculations are too computationally exhaustive, leading to UCA3.

**Table 8:** Some results related to the autonomous navigation system from the human-oriented STPA addressing different LoA, from [67].

CA	Type of UCA	UCA
Provide an updated trajectory	Not provided	The autonomous navigation system does not provide the designated route.
	Provided incorrectly or when not needed	The autonomous navigation system provides an updated trajectory when collision risk exists.
	Provided too early or too late	The autonomous navigation system provide the designated route too late.
	Provided too early	The autonomous navigation system provides an updated trajectory too late when collision risk exists.

These examples are included here to show the difference in completeness with respect to software failures in STPA. The results from Table 7, Table 8 are valuable given the scope of these analysis, e.g., for defining high level safety requirements, but they are much less detailed with respect to identifying, analyzing, and preventing software failures (cf. Table 5), and, as such, for providing useful information in the design process of safe software for autonomous ships.

In a more conventional risk analysis, using PHA and a bayesian belief network (BBN), Guo et al. [68] assess the collision risk of an autonomous ferry's passage in the Trondheim channel. Obstacle detection function failure, cyber attack, and decision system failure were found to be sensitive nodes in the analysis. These failures are related to software failures analyzed in this paper, namely SF-05, SF-06, SF-07, and SF-08 (Table A.9), covering false positive and false negative static and moving obstacle detection events.

### 5.3 Compatibility of STPA SW-SAF-SEC with other STPA approaches

The STPA SW-SAF-SEC approach presented in this paper is compatible with other STPA methods, even though it strongly focuses on SW. In particular, the following methodological aspects can be highlighted:

*Control structure*

The classical STPA often considers controllers as logical units that can be mapped back to the system's functional components. STPA SW-SAF-SEC, however, defines three functional controller types for HO, HW, and SW and their mutual interfaces. The logical controller can be any of the mentioned individual types or a mixed-type controller, e.g., the HO-SW user interface covering both HO interactive actions and SW response computation.

The software function, the software part of the HO-SW interface, and the software part of the HW-SW interface use an extended modeling approach to cover the necessary details for safety analysis. The STPA analyst determines where to model the internal controller's VAR and dynamic behavior, but this does not constrain the type of controller and its realization (see Table 2). Also, the control structure is still compatible with classic STPA.

*Dynamic interactions*

The classical STPA encapsulates the system functions in its controllers and emulates dynamic interactions utilizing CAs. The system's hierarchical and dynamic evolution is modeled as an “event sequence” of CA calls between controllers. The dynamic sequence can be initialized by any controller or periodic event. In STPA SW-SAF-SEC, the control hierarchy is extended by adding VFs, modeled as dynamic interactive activities with the evolution of the controller state and the VARs' values. This extension creates an environment that enables the examination of the inner workings of the software and the detection of possible software failures. Possible software failures may lead to safe and UCAs, which is similar to other STPA approaches.

*Loss scenarios*

The loss scenarios in classical STPA include UCAs influencing the dynamic state of a system, leading to hazards and losses. STPA SW-SAF-SEC also includes software failure events that, e.g., may lead to UCAs. The dynamic state evolution is extended by a runtime variable evolution, which adds additional information to the STPA control hierarchy. The VAR value evolution impacts the development of the loss scenarios, like following alternative flows in the software function. This additional information enables the development of more precise loss scenarios, but the approach should still be compatible with the classical STPA since the software failures and the runtime VAR evolution are extensions.

To summarize, since STPA SW-SAF-SEC follows the classical STPA process at an overall level, it is possible to combine it with analyses focused on human errors, and HW failures, etc. Then the more regular steps of STPA, e.g., related to identification of UCAs, can be followed in parallel.

### 5.4 Applying the results of the STPA SW-SAF-SEC approach to quantitative risk modeling

In the STPA SW-SAF-SEC method, both dynamic and static parts of the control hierarchy are created providing useful insights into the inner workings of software failures, hazardous events, and potential losses. The approach therefore provides an extensive and systematic foundation for further quantitative risk assessment and modeling, online risk assessment, dynamic probabilistic risk assessment (DPRA), and supervisory risk control (SRC); provided that the necessary data is available.

A relevant example is that software failures may lead to different consequences, as indicated in the loss scenarios. It may be desirable to investigate such consequences more in detail, which can be done using event tree analysis (ETA). Utne et al. [69] use the results of STPA to develop a BBN model for online risk assessment and SRC, including UCAs and information from the loss scenarios as part of the nodes in the BBN. The loss scenarios may aid in the risk quantification of the risk influencing factors (RIFs) or nodes in the BBN leading to more realistic results. The resulting BBN may contain software failure-related nodes on the high system level, which can be more detailed if STPA-SW-SAF-SEC is used as the underlying method.

Yang et al. [70] have modeled online risk assessment for AUVs operating under the ice. The proposed BBN for online risk assessment can be further extended by control software failure when an AUV is operating in autonomous mode during the transit period between buoys.

SRC is an approach used for real-time decision-making (see e.g., [69], [71]) where the operational conditions, AMS performance parameters, and predictions are linked in a BBN risk model to calculate the online or future risk of losses. The output of the BBN is processed by the control system to make safe decisions. SRC may involve both actual and future risk estimates of AMSs during operation. These are emerging applications that may have a significant impact on system safety and security during operation.

DRPA utilizes an event sequence of discrete bounded events to predict possible future hazards and losses. The loss scenarios created in STPA SW-SAF-SEC are sequences of discrete bounded events leading to hazardous events resulting in system-level losses. STPA SW-SAF-SEC provides a qualitative risk assessment and description of software failure events. If the software failure's event frequency data can be obtained via testing or another statistical method, then the software failure event can be used as random events in DPRA scenario generation.

### 5.5 Limitations and further work

The case study in the paper shows the possibility of identifying a large volume of software failures and later evaluating their significance qualitatively in loss scenarios. The loss scenarios are situation-dependent and defined according to the AMS's expected operation. It may become quite cumbersome and resource demanding to perform a detailed analysis of all software failures and loss scenarios. Hence, there is a need to determine a software failure's significance within loss scenarios to reduce the overall number of software failures and efforts on implementing redundant countermeasures. In addition, a loss scenario generation method would be beneficial independent of operational context to identify potential software failures that may occur regardless of operational context.

More extended quantitative risk analysis may help prioritizing risk reduction measures, which can be challenging when considering the qualitative results of the case study. This is, however, also a challenge with the “classical” STPA, i.e., that the qualitative analysis may become too resource demanding in terms of identifying sufficient risk reduction and countermeasures. A quantitative analysis of the software failures' frequency, magnitude, and evaluated causality is omitted in MarCrew case study in the paper, as the input from the CONOPS is valid for operational and system levels, and relevant quantitative data for a similar operation could not be retrieved.

SIL and HIL are used for testing, verification and validation. The proposed approach in the paper is qualitative of nature, which means that more information and work is needed in order to develop a quantitative foundation for such types of analyses. In the case study, a quantitative risk analysis of the software failures is missing due to lack of failure and risk data. Such types of data is a challenge to collect for different abstraction levels and operations.

Software specialists are required to be involved in the analysis, similar to the involvement of other types of expertises in an STPA. Nevertheless, the software failure categorization and the GQs also provide support for non-software engineers to be able identify and investigate potential problems in software and aid in the development of safe systems.

STPA SW-SAF-SEC may be performed by product or system development team members with the necessary expertise. Detailed UML and SysML modeling may be challenging in the early system development stage and thus often needs to be included in existing projects. Similarly to other types of hazard and risk analysis, the teamś expertise, preferences, knowledge and experience may influence the results of the analysis.

Further work should focus on the following issues:

• The loss scenarios developed in the paper are qualitative, and risk quantification has not been implemented in the proposed method. This should be subject to further work.
• AMS use the safety integrity level (SIL) concept that requires software designers to achieve system-specific failure rates and impact magnitudes at the system level. The software parts contribute to the overall system's failure, so their parameter quantification is necessary for further validation and verification. Hence, further work should focus on integration with simulation for quantitative risk modeling and validation.
• Further work should also be performed with respect to investigating the feasibility of the approach to other application areas than AMS.
• The proposed approach is limited to UML and SysML deployment and sequence diagrams. While utilizing other static and dynamic models could be possible, it has yet to be practically demonstrated.
• In this paper, we have not included UCA related to HO, but this could be included, e.g., by following the approach as suggested by Cheng et al. [67]. This should also be subject to further work.

## 6 Conclusion

This paper presents the STPA SW-SAF-SEC approach, which is an extension of STPA, focusing on identifying and analyzing software failures and their corresponding UCAs and loss scenarios. The results may contribute to more cost-efficient and safer software design if the method is performed early in the system design and development phase. The results may also provide a foundation for further development of online risk assessments, DPRA and SRC to achieve more intelligent autonomous systems, or provide a basis for other types of quantitative risk assessments for operational decision support.

The approach includes enhanced modeling of the STPA control hierarchy and identification of potential software failures. The control hierarchy structure in the proposed approach includes the use of SysML and UML models which enables analysis at the operational to implementation abstraction levels during the AMS's development life cycle.

The important software functions are decomposed into VARs and interaction models, enabling the software parts to cover the complex runtime evolution. The inclusion of the explicit internal state and VARs to the controllers covering software parts facilitates the search for potential software failures. Software failures may lead to system-level UCAs, causing hazards and losses.

GQs are included to help identify software failures, and these reflect the potential impact of seven major software failure categories which apply to a wide range of system architectures and abstraction levels. Significant software failures may be identified by their impact evaluation in specific operational situations through loss scenarios. The hazard mitigation of software failures is done by applying a combination of various traditional and innovative techniques. The main contribution to hazard mitigation is introducing fallback procedures that maintain normal system function in case of software failure.

In the paper, the presented STPA SW-SAF-SEC has been applied to a ferry's navigation system switching between manual and semi-autonomous operating modes during transit. The case study identified significant software failures which potentially may contribute to catastrophic losses like collision, allision, and grounding. One of the loss scenarios shows that this system cannot operate with worsened visibility for a given mission, and that clear port-to-port visibility is required for the autonomous transit. The proposed hazard mitigation shows that the potential impact of software failures on the overall system risks can be reduced in the early system development, lowering the development and testing costs and improving safety and security in operation.

## CRediT authorship contribution statement

**Alojz Gomola:** Writing – original draft, Visualization, Methodology, Investigation, Formal analysis, Data curation, Conceptualization. **Ingrid Bouwer Utne:** Writing – review & editing, Validation, Supervision, Resources, Project administration, Funding acquisition, Data curation, Conceptualization.

## Declaration of Competing Interest

The authors declare that they have no known competing financial interests or personal relationships that could have appeared to influence the work reported in this paper.

## Figures and Tables

**Table A.9:** The Identified Software Failures.

SFC	GQ	ID	OVF	Step	Name		
REX	GQ-01	SF-01	VF-08	S.2.7	Large [VAR-07] Active Avoidance Map	**Cause:**	**Consequences:**
						S.2.7 Returns too many Tracked Targets	[UCA] Unavailable [VF-08] Predict Future Tracks of Targets
							Invalid, Incorrect [1..1][VAR-23] Active Avoidance Map
						**Trace:**	**Recoverability:**
						S.2.11 Predicts for all Tracked Targets	Yes - the next scheduled run of [VF-06],
						S.2.12 Projects trajectories into Avoidance Map	If [0..*][VAR-06] Tracked Targets changes
	GQ-02	SF-02	VF-08	S.2.11	Heavy computation of Predicted Trajectories	**Cause(s):**	**Consequences:**
						S.2.7 [CA-23] Returns many [VAR-06] Tracked Targets	[UCA] Unavailable [VF-08] Predict Future Tracks of Targets
						S.2.11 Implementation has exponential scaling	[UCA] Too late [VF-08] Predict Future Tracks of Targets
							[1..1]Invalid, Incorrect [VAR-23] Active Avoidance Map
						**Trace:**	**Recoverability:**
						S.2.11 Exhaust computation capacity of [CS-04]	Yes - the next scheduled run of [VF-06],
						S.2.11 Takes too long or fails	If [0..*][VAR-06] Tracked Targets changes
	GQ-04	SF-03	VF-08	S.2.8	Memory Fragmentation [CS-04] caused by [VF-08]	**Causes:**	**Consequences:**
						S.2.1 Scheduled Run (...) called periodically	Memory fragmentation [CS-04]
						S.2.7 (Re)allocates [VAR-06] and [VAR-11]	[UCA] Unavailable [CA-37] Fetch Viable Alternative Routes
							[UCA] Unavailable [CA-57] Fetch Active Collision Risk Warnings
							Valid, incorrect [VAR-25] Active Collision Risk Warning
						**Trace:**	**Recoverability:**
						S.2.8 [0..*][VAR-06] Tracked Targets	No - the [CS-04] state is permanent
						Frequent allocation of [0..*][VAR-06]	
						S.2.10 [0..*][VAR-11] Snapshot Tracked Targets	
						Frequent relocation of [0..*][VAR-11]	
	GQ-08	SF-04	VF-08	S.2.8	Unconstrained [VAR-06] Tracked Targets	**Causes:**	**Consequences:**
						[CS-02] can process up to 100 tracked targets	[CS-02] is unable to display all Tracked Targets
						S.2.8 Returns [0..*][VAR-24] Tracked Targets	Valid, correct, inconsistent [VAR-19] Avoidance Map
						Count of [VAR-24] > 100	
						**Trace:**	**Recoverability:**
						S.2.11 Predicts trajectories for all [VAR-06]	Yes - If count of [VAR-24] <=100
						S.2.12 Projects all trajectories into [VAR-19]	
						[VAR-19] contains all Tracked Targets	

BEH	GQ-10	SF-05	VF-08	S2.12	Nonexistent Moving Obstacle trajectory projection	**Causes:**	**Consequences:**
						S.2.7 [CA-24] Fetch Tracked Targets	[UCA] Invalid Collision Risk in [VF-06] for Phantom Ship
						Valid, Inconsistent [VAR-06]Tracked Targets	Valid, correct, inconsistent [VAR-19] Avoidance Map
						**Trace:**	**Recoverability:**
						S.2.8 Returns phantom Tracked Target	Yes - If Phantom Tracked Target is not provided next
						S.2.11 Predicts its trajectory	run of [VF-08]
						S.2.12 Projects trajectory into Avoidance Map	
		SF-06	VF-08	S.2.15	Nonexistent Static Obstacle projection	**Causes:**	**Consequences:**
						S.2.14 returns outdated [VAR-18] Current Chart	[UCA] Invalid Static Obstacle in [VF-06] form [VAR-18]
						Outdated [VAR-18] Current Chart	Valid, correct, inconsistent [VAR-19] Avoidance Map
						Nonexistent Static Obstacles present	
						**Trace:**	**Recoverability:**
						S.2.13 Successful in the operational area	Yes - If a Nonexistent Static Obstacle is not
						S.2.14 Returns outdated [VAR-18]	provided next run of [VF-08]
						S.2.15 Projects nonexistent Static Obstacle	
	GQ-11	SF-07	VF-08	S.2.12	Omitted Moving Obstacle trajectory projection	**Causes:**	**Consequences:**
						S.2.7 [CA-24] Fetch Tracked Targets	[UCA] Omitted in [VF-06] Determine Collision Risk of Targets
						Valid, Inconsistent [VAR-06]Tracked Targets	Valid, correct inconsistent [VAR-19] Avoidance Map
						**Trace:**	**Recoverability:**
						S.2.8 Omits existing Tracked Target	Yes - If Tracked Target is provided next run of [VF-08]
						S.2.11 Omits its trajectory	
						S.2.12 Omitted projection of trajectory	
		SF-08	VF-08	S.2.15	Omitted Static Obstacle projection	**Causes:**	**Consequences:**
						S.2.14 returns outdated [VAR-18] Current Chart	[UCA] Omitted Static Obstacle in [VF-06] form [VAR-18]
						Outdated [1..1][VAR-18] Current Chart	Valid, correct, inconsistent [VAR-19] Avoidance Map
						Uncharted Static Obstacle	
						**Trace:**	**Recoverability:**
						S.2.13 Successful in the operational area	Yes - If the existing Static Obstacle is provided
						S.2.14 Returns outdated [VAR-18]	next run of [VF-08]
						S.2.15 Omits existing Static Obstacle	

SYN	GQ-15	SF-09	VF-08	S.2.15	Exceeding timestamp difference between time-sensitive variables	**Causes:**	**Consequences:**
						Timestamp of [VAR-07] Freespace Map	[UCA] Inconsistent [VF-08] Predict Future Tracks of Targets
						Timestamp of [VAR-06] Tracked Targets	Valid, incorrect, inconsistent [VAR-19] Avoidance Map
						Timestamp of [VAR-18] Current chart	
						Exceeded timestamp validity difference	
						**Trace:**	**Recoverability:**
						S.2.4 Fetches [VAR-07]	Yes - if the next scheduled run obtains correct
						S.2.6 Projects [VAR-07] into [VAR-19]	[VAR-07], [VAR-06], and [VAR-18]
						S.2.7.Fetches [VAR-06]	
						S.2.11 Predict trajectories for [VAR-06]	
						S.2.12 Projects trajectories to [VAR-19]	
						S.2.13 Fetches [VAR-18]	
						S.2.15 Projects [VAR-18] to [VAR-19]	
	GQ-16	SF-10	VF-08	S.2.11	Inconsistent trajectory prediction caused by Tracked Targets Snapshots	**Causes:**	**Consequences:**
						Valid [VAR-06] Tracked Target	[UCA] Inconsistent [VF-08] Predict Future Tracks of Targets
						Valid, Inconsistent [VAR-11] Snapshot Tracked Targets	Valid, correct, inconsistent [VAR-19] Avoidance Map
						**Trace:**	**Recoverability:**
						S.2.8 Returns [0..*][VAR-06]	Yes - If correct, [VAR-06] is provided next
						S.2.10 Returns [0..1] [VAR-11]	run of [VF-08]
						S.2.11 Predicts future trajectories incorrectly	
						S.2.12 Projects future trajectories correctly	

DDE	GQ-20	SF-11	VF-08	S.2.4	Data degradation of Freespace Map	**Cause:**	**Consequences:**
						Invalid [VAR-07] Freespace Map	[UCA] Failed [VF-08] Predict Future Tracks of Targets
							Invalid [VAR-19] Avoidance Map
						**Trace:**	**Recoverability:**
						S.2.5 Returns invalid [VAR-07]	Yes - If valid [VAR-07] is provided next
						S.2.6 Unable to project [VAR-07]	run of [VF-08]
						S.2.16 Failed update [VAR-19] Avoidance map	
		SF-12	VF-08	S.2.7	Data degradation of Tracked Targets	**Cause:**	**Consequences:**
						Invalid [VAR-06] Tracked Targets	[UCA] Failed [VF-08] Predict Future Tracks of Targets
							Invalid [VAR-19] Avoidance Map
						**Trace:**	**Recoverability:**
						S.2.7 Returns invalid [VAR-06]	Yes - If valid [VAR-06] is provided next
						S.2.11 Failed trajectory predictions	run of [VF-08]
						S.2.12 Failed trajectory projection	
						S.2.16 Failed update [VAR-19] Avoidance Map	
		SF-13	VF-08	S.2.13	Data degradation of Current Chart	**Cause:**	**Consequences:**
						Invalid [VAR-18] Current Chart	[UCA] Failed [VF-08] Predict Future Tracks of Targets
							Invalid [VAR-19] Avoidance Map
						**Trace:**	**Recoverability:**
						S.2.14 Returns invalid [VAR-18]	Yes - If valid [VAR-18] is provided next
						S.2.15 Failed Static Obstacles projection	run of [VF-08]
						S.2.16 Failed update [VAR-19] Avoidance Map	
	GQ-24	SF-14	VF-08	S.2.7	The mismatch between Fetched Tracked Target and Snapshot	**Causes:**	**Consequences:**
						Valid [VAR-06] Tracked Target	[UCA] Inconsistent [VF-08] Predict Future Tracks of Targets
						Invalid [VAR-11] Snapshot Tracked Targets	Valid, incorrect, inconsistent [VAR-19] Avoidance Map
						The mismatch between [VAR-06] and [VAR-11]	
						Unable to use past position from [VAR-11]	
						Hard to predict the future position for [VAR-06]	
						**Trace:**	**Recoverability:**
						S.2.7 Fetch online [VAR-06]	Yes - If all snapshots of [VAR-06] are replaced
						S.2.9 Loads stored [VAR-11]	with correct ones
						S.2.11 Fails to predict trajectories correctly	
						S.2.12 Projects incorrect future trajectories	
						S.2.16 Updates [VAR-19] Avoidance Map	

CON	GQ-27	SF-15	VF-08	S.2.13	Chart data format due to [CS-02] swap	**Causes:**	**Consequences:**
						[CS-02] Map data format change	[UCA] Failed [VF-08] Predict Future Tracks of Targets
						Invalid [VAR-18] Current Chart	Invalid [VAR-19] Avoidance Map
						**Trace:**	**Recoverability:**
						S.2.13 Fetch [VAR-18]	No, there is no immediate recovery option
						S.2.14 Returns invalid [VAR-18]	during system operation
						S.2.15 Failed projection of Static Obstacles	
						S.2.16 Failed update [VAR-19] Avoidance Map	
	GQ-28	SF-16	VF-08	S.2.4	No communication due to SSL Library change in [CS-02],[CS-01]	Causes:	**Consequences:**
						SSL Library changed in [CS-02],[CS-01]	[UCA] Failed [VF-08] Predict Future Tracks of Targets
						Unable to fetch data from [CS-01]	Invalid [VAR-19] Avoidance Map
						Unable to fetch data from [CS-02]	
						**Trace:**	**Recoverability:**
						S.2.4 SSL Handshake failure	No, there is no immediate recovery option
						S.2.6 Unable to project [VAR-07]	during system operation
						S.2.7 SSL Handshake failure	
						S.2.12 Unable to project trajectories	
						S.2.13 SSL Handshake failure	
						S.2.15 Unable to project Static Obstacles	
						S.2.16 Failed update [VAR-19] Avoidance Map	
	GQ-29	SF-17	VF-08	S.2.7	Tracked Targets imprecision due to Sensor Array change	**Causes:**	**Consequences:**
						[CS-07] SINT replaced	[UCA] Imprecise [VF-08] Predict Future Tracks of Targets
						[CS-01] Sensor fusion valid, imprecise	Valid, correct, imprecise [VAR-19] Avoidance Map
						Imprecise [VAR-06] Tracked Targets	
						**Trace:**	**Recoverability:**
						S.2.7 Fetch [VAR-06]	No, there is no immediate recovery option
						S.2.11 Imprecise trajectory prediction	during system operation
						S.2.12 Proper trajectory projection	
						S.2.16 Imprecise [VAR-19] Avoidance Map	

SEC	GQ-30	SF-18	VF-08	S.2.13	Malicious change of Active Avoidance Map	**Causes:**	**Consequences:**
						Breach to [CS-04] Volatile memory	[UCA] Manipulated [VF-08] Predict Future Tracks of Targets
						Manipulation of stored [VAR-23] Active Avoidance Map	Valid, manipulated, imprecise [VAR-19] Avoidance Map
						Successful update of [VAR-19] Avoidance Map	[Hazard] Collision, allision, grounding
						**Trace:**	**Recoverability:**
						S.2.13 Changed [VAR-23] loaded	No, the successful data manipulation
						S.2.16 Update [VAR-19] successful	cannot be recovered
	GQ-33	SF-19	VF-08	S.2.7	Phantom Ship insertion into [VAR-06] in [CS-01]	**Causes:**	**Consequences:**
						Data manipulation [VAR-06] Tracked Targets	[UCA] Invalid Collision Risk in [VF-06] for Phantom Ship
						Moving Obstacles inserted/changed	Valid, correct, inconsistent [VAR-19] Avoidance Map
						**Trace:**	**Recoverability:**
						S.2.7 Manipulated [VAR-06] requested	No, the successful data manipulation
						S.2.11 Falsified trajectories predicted	cannot be recovered
						S.2.12 Falsified trajectories projected	
						S.2.16 Falsified [VAR-19] Avoidance Map	

CAS	GQ-35	SF-20	VF-06	S.3.2	Unavailable Avoidance Map for [VF-06]	**Causes:**	**Consequences:**
						SF-01, SF-02, SF-03, SF-11, SF-12,	[UCA] Failed [VF-06] Determine Collision Risk of Targets
						SF-13, SF-15, SF-16	Valid, Incorrect [VAR-25] Active Collision Risk Warnings
							[Hazard] Collision, allision, grounding
						**Trace:**	**Recoverability:**
						S.3.2 Unable to load [VAR-23]	Yes - if causing SF
						S.3.12 No Risk for Moving Obstacles calculated	recovers in the next [VF-06] run
						S.3.13 No Risk for Static Obstacles calculated	
						S.3.17 No [VAR-25] Active Collision Risk Warnings	
		SF-21	VF-06	S.3.2	Nonexistent Moving Obstacle processed in [VF-06]	**Causes:**	**Consequences:**
						SF-05, SF-09, SF-10, SF-14, SF-17,	[UCA] Corrupted [VF-06] Determine Collision Risk of Targets
						SF-18, SF-19	Valid, Incorrect [VAR-25] Active Collision Risk Warnings
							[Hazard] Collision, allision, grounding
						**Trace:**	**Recoverability:**
						S.3.2 Corrupted [VAR-23] loaded	Yes - if causing SF
						S.3.12 Incorrect Risk with Moving Obstacle calculated	recovers in the next [VF-06] run
						S.3.14 Incorrect Thresholding	
						S.3.18 False Positive [VAR-25] stored	
		SF-22	VF-06	S.3.2	Nonexistent Static Obstacle processed in [VF-06]	**Causes:**	**Consequences:**
						SF-06, SF-18	[UCA] Corrupted [VF-06] Determine Collision Risk of Targets
							Valid, Incorrect [VAR-25] Active Collision Risk Warnings
							[Hazard] Collision, allision, grounding
						**Trace:**	**Recoverability:**
						S.3.2 Corrupted [VAR-23] loaded	Yes - if causing SF
						S.3.13 Incorrect Risk with Static Obstacle calculated	recovers in the next [VF-06] run
						S.3.14 Incorrect Thresholding	
						S.3.18 False Positive [VAR-25] stored	
		SF-23	VF-06	S.3.2	Omitted Moving Obstacle in [VF-06]	**Causes:**	Consequences:
						SF-07, SF-09, SF-10, SF-14, SF-17,	[UCA] Corrupted [VF-06] Determine Collision Risk of Targets
						SF-18, SF-19	Valid, Incorrect [VAR-25] Active Collision Risk Warnings
							[Hazard] Collision, allision, grounding
						**Trace:**	Recoverability:
						S.3.2 Corrupted [VAR-23] loaded	Yes - if causing SF
						S.3.12 Incorrect Risk with Moving Obstacle calculated	recovers in the next [VF-06] run
						S.3.14 Incorrect Thresholding	
						S.3.18 False negative [VAR-25] stored	
		SF-24	VF-06	S.3.2	Omitted Static Obstacle in [VF-06]	**Causes:**	**Consequences:**
						SF-08, SF-18	[UCA] Corrupted [VF-06] Determine Collision Risk of Targets
							Valid, Incorrect [VAR-25] Active Collision Risk Warnings
							[Hazard] Collision, allision, grounding
						**Trace:**	**Recoverability:**
						S.3.2 Corrupted [VAR-23] loaded	Yes - if causing SF
						S.3.13 Incorrect Risk with Static Obstacle calculated	recovers in the next [VF-06] run
						S.3.14 Incorrect thresholding	
						S.3.18 False negative [VAR-25] stored	

## Appendix A Identified software failures in MarCrew

This appendix contains the identified software failures for [VF-08] Predict Future Tracks of Targets actions (Fig. 5) and [VF-06] Determine Collision Risk of Targets (Fig. 6).

The Table A.9 contains the following columns, further describing the identified software failures:

1. Software Failure Category (SFC) - defines the type of software failure category that has been assigned according to the GQ (Table 1).
2. Guiding question (GQ) - that has been triggered during the software failure search on control hierarchy views (see Table 1).
3. Identification number (ID) - a unique identifier of software failure used in loss scenarios (Table 5) and hazard elimination and mitigation (Table 6).
4. Origin Vessel Feature (OVF) - the vessel feature identifying the software failure's origins; refer to the specific sequence diagram of VF.
5. Step - the step of the sequence diagram model of VF, where the software failure begins its manifestation; for more details, see the cause.
6. Name - the software failure's name reflects the cause, trace, and consequences in shorthand.
7. Cause - the cause of software failure, covering both static and dynamic aspects.
8. Trace - the trace of the software failure from the starting step to the VF's sequence diagram boundary, including step mapping and deviations from expected behavior.
9. Consequence - the ultimate consequence of the software failure at the boundary of VF or CS, listing UCAs, hazards, losses, and changes to dynamic control hierarchy runtime VARs, CAs, and VFs.
10. Recoverability - indicates the possibility of software failure recovery and lists conditions under which it will recover.

## Footnotes

1 UML sequence diagrams reference. https://developer.ibm.com/articles/the-sequence-diagram/
